# Single-cell RNA sequencing identifies ZBP1-dependent mechanisms in OSCC progression

**DOI:** 10.1038/s41419-025-08349-7

**Published:** 2025-12-22

**Authors:** Xuyang Lin, Chenlong Wang, Chaoyang Li, Jin Wu, Ping Zhang, Chengwan Zhang, Hao Chen, Fangyi Xu, Shuangyue Zhang, Chao Luo, Chunbo Tang

**Affiliations:** 1 Department of Oral Implantology, The Affiliated Stomatological Hospital of Nanjing Medical University, Nanjing, Jiangsu Province China; 2 Department of Central Laboratory, The Affiliated Huaian No.1 People’s Hospital of Nanjing Medical University, Northern Jiangsu Institute of Clinical Medicine, Nanjing Medical University, Nanjing, Jiangsu Province China; 3 Jiangsu Province Engineering Research Center of Stomatological Translational Medicine, Nanjing Medical University, Nanjing, Jiangsu Province China; 4 State Key Laboratory Cultivation Base of Research, Prevention and Treatment for Oral Diseases, Nanjing Medical University, Huai’an, Jiangsu Province China; 5 Department of Oral and Maxillofacial Surgery, The Affiliated Huaian No.1 People’s Hospital of Nanjing Medical University, Huaian, Jiangsu Province China

**Keywords:** Oral cancer, Oncogenes

## Abstract

Oral squamous cell carcinoma (OSCC) is a highly aggressive head and neck malignancy with a poor prognosis associated with its complex tumor microenvironment. Cancer-associated fibroblasts (CAFs) contribute to tumor progression by secreting various signaling molecules. This study investigates the molecular mechanism through which Z-DNA-binding protein 1 (ZBP1) promotes OSCC development through CAF regulation. To this end, orthotopic MOC1 transplantation and 4NQO-induced carcinogenesis OSCC models were established with *Zbp1*^−/−^ mice. Single-cell RNA sequencing (scRNA-seq) analyzed cellular heterogeneity and signaling network alterations in the tumor microenvironment. An in vitro CAF induction model combined with a Transwell co-culture system clarified the molecular mechanism of ZBP1. Finally, the role of the ZBP1–CCL7/CCR1 signaling axis in promoting OSCC progression was evaluated via in vivo recombinant CCL7 protein rescue and CCR1 antagonist (BX471) intervention. ZBP1 is highly expressed in OSCC tissues, while its deficiency inhibits tumor growth and proliferation. Proliferation-related pathways (e.g., E2F targets, MYC targets, cell cycle) are downregulated while immune activation signatures (e.g., interferon response, p53 pathway, TNF-α/NF-κB signaling) are upregulated in *Zbp1*^−/−^ tumor cells. Cellular interaction analysis and ligand–receptor network profiling demonstrated significant attenuation of the CCL7–CCR1 signaling axis between CAFs and tumor cells. ZBP1 deficiency reduces CCL7 expression in CAFs, diminishing their ability to promote tumor cell proliferation, migration, and invasion via the CCL7/CCR1 axis. Exogenous CCL7 supplementation partially restores tumor growth in *Zbp1*^−/−^ mice, indicating that ZBP1 bridges CAF–tumor cell communication through the CCL7–CCR1 axis. This study highlights ZBP1 as crucial for OSCC progression by regulating CCL7 expression in CAFs to activate CCR1 signaling in tumor cells. This provides insights into the regulatory mechanisms within the OSCC microenvironment, offering a potential therapeutic strategy for targeted interventions.

## Introduction

Oral squamous cell carcinoma (OSCC) is a highly aggressive head and neck squamous cell carcinoma (HNSCC), accounting for approximately 90% of cases [1]. Despite advancements in diagnostics and precision therapies, the 5-year survival rate remains at 40–50% [2]. The poor prognosis is due to its intrinsic propensity for metastasis, recurrence, and resistance to treatment, largely attributable to the complex tumor microenvironment (TME) and interplay between tumor and surrounding stromal cells. Cancer-associated fibroblasts (CAFs)—the most abundant stromal component in the TME—drive tumor progression by remodeling the extracellular matrix (ECM), secreting cytokines (e.g., interleukin [IL]-6 and transforming growth factor [TGF]-β), and activating key pathways (e.g., Wnt/β-catenin, Hedgehog) to confer stem-like properties to tumor cells [3]. CAFs also regulate the expression of immune checkpoints (e.g., PD-L1/CTLA-4), promoting an immune-evasive microenvironment [4]. Notably, CAF heterogeneity in HNSCC correlates with disease progression, supporting stroma-targeted therapies [5].

Z-DNA-binding protein 1 (ZBP1) is a pattern recognition receptor characterized by its conformation-specific recognition of left-handed Z-nucleic acid conformations, including Z-DNA and Z-RNA, through its two highly conserved Zα domains. It exerts dual roles in pathogen recognition and tumor immunomodulation[6]. Additionally, the C-terminal RIP homotypic interaction motif (RHIM) of ZBP1 forms signaling complexes with receptor-interacting protein kinases (RIPK1/3), initiating programmed cell death cascades [7]. It also promotes the maturation and release of inflammatory mediators (e.g., IL-1β/IL-18) by activating the NLRP3 inflammasome-caspase-1 axis, ultimately triggering PANoptosis—a newly defined form of inflammatory programmed cell death that integrates features of pyroptosis, apoptosis, and necroptosis (hence the term ‘PANoptosis’) [8]. Collectively, ZBP1 broadly influences the TME by regulating stromal cell functions, inflammatory responses, immune evasion, and cell death pathways, thereby promoting tumor initiation, progression, and metastasis [9]. Although existing studies suggest that ZBP1 participates in the pathogenesis of HNSCC, its specific role in OSCC is unclear, necessitating further investigation [10].

This study aimed to elucidate the regulatory role of ZBP1 in OSCC progression. To this end, *Zbp1*^−/−^ (whole-gene knockout) OSCC mouse models, single-cell transcriptomic sequencing, and functional gain/loss-of-function experiments were employed. This study is the first to reveal the tumor-promoting function of ZBP1 in OSCC and delineate the specific mechanistic axes in CAF–tumor cell interactions within the TME, providing a theoretical foundation and potential targets for OSCC treatment.

This discovery underscores the critical role of chemokine signaling networks in stromal–tumor cell crosstalk [11–13]. CCL7 (monocyte chemoattractant protein 3, MCP-3) was initially isolated and identified in osteosarcoma cell-conditioned medium [14]. Secreted by monocytes, fibroblasts, platelets, and tumor cells, CCL7 binds to its cognate receptors to regulate immune cell chemotaxis, inflammatory responses, and tumor progression [15]. Its primary receptor CCR1 is a G protein-coupled receptor widely expressed in immune cells that recognizes ligands (CCL3, CCL5, and CCL7) that mediate downstream signaling to modulate cell migration and activation [16, 17]. While existing studies have confirmed the involvement of this signaling axis in metastasis, TME remodeling, and immune evasion across multiple solid tumors, its specific role in OSCC, particularly in CAF–tumor cell communication, remains undefined [18–20].

## Materials and Methods

### Cell culture

The mouse oral squamous cell carcinoma cell line MOC-1 was procured from Kerafast (USA) and maintained in DMEM/F12 medium (Gibco, Thermo Fisher, USA), supplemented with 10% fetal bovine serum (FBS, Newzerum, New Zealand), 1% penicillin/streptomycin (Invitrogen, Thermo Fisher), 10 ng/mL epidermal growth factor (MedChemExpress, USA), 10 μg/mL insulin (MedChemExpress, USA), and 80 ng/mL hydrocortisone (MedChemExpress, USA). The culture was conducted at 37 °C in a humidified incubator with 5% CO₂. Mouse embryonic fibroblasts (MEFs) were acquired from Cyagen Biosciences (China) and cultured in DMEM medium (Gibco, Thermo Fisher, USA) containing 10% FBS and 1% penicillin/streptomycin under identical conditions.

For the in vitro pharmacological experiments, cells were maintained under standard culture conditions and subjected to treatment with either BX471 at a concentration of 10 μM (MedChemExpress, USA) or recombinant mouse CCL7 protein at 100 ng/mL (MedChemExpress, USA) as described in refs. [21, 22]. Control groups were administered vehicle treatments, consisting of either PBS or DMSO.

### CAFs Induction

Tumor-conditioned medium (TCM) was prepared based on previously described methods [23]. MOC-1 cells were cultured in medium containing 2% FBS for 48 h, and the supernatant was collected, centrifuged at 1000 rpm for 5 min, and stored at −20 °C. MEFs were co-cultured with TCM for 72–96 h. The induction of CAFs was validated by RT-qPCR.

### *Zbp1* Knockout Mice and OSCC Model Establishment

*Zbp1* knockout (*Zbp1*^−/−^) mice were purchased from Cyagen Biosciences (Suzhou, China) (Supplemental Fig. 1). Genotyping primer sequences are listed in supplementary Table 1. OSCC models were established using two approaches: [1] Cell injection model: MOC-1 cells (2.0 × 10^5^ in 40 µL PBS) were injected into the lateral tongue of both *Zbp1*^−/−^ and wild-type (WT) mice (ten mice each group). Tumor length and width were measured every 3–5 days from day 14, and tumor volume was calculated as (length × width²)/2. Mice were euthanized when tumor volume reached ≥150 mm^3^ or body weight decreased by ≥25%, and tumors were harvested for further analysis [2]. Chemical induction model: 4-nitroquinoline 1-oxide (4NQO, 100 mg/L, Sigma, USA) was added to the drinking water for 16 weeks, followed by regular water until week 24, six mice each group were euthanized. Tumor measurements and tissue collection were performed as described above [24]. For pharmacological interventions, starting on day 7 after MOC-1 injection, mice received intraperitoneal injections of BX471 (50 mg/kg, QD) and recombinant CCL7 protein (100 μg/kg, QD) for three consecutive weeks [25–27]. All mice were euthanized at week 3 for tumor tissue collection. This study was approved by the Ethics committee of Huai’an No. 1 People’s Hospital and was conducted in accordance with the guidelines of the National Animal Care and Ethics Institution.

### ZBP1 expression analysis using public datasets

The expression of ZBP1 in human tumor and normal tissues was examined utilizing web-based analytical tools and microarray datasets. Specifically, Gene Expression Profiling Interactive Analysis 2 (GEPIA2) was employed to conduct a comparative analysis of ZBP1 expression levels between head and neck squamous cell carcinoma (HNSCC) samples obtained from The Cancer Genome Atlas (TCGA) and corresponding normal tissues sourced from the Genotype-Tissue Expression (GTEx) database. The analysis was performed utilizing the “Expression DIY” module with its default parameters. Furthermore, two datasets from the Gene Expression Omnibus (GEO), specifically GSE37991 and GSE30784, were employed to further investigate the differential expression of ZBP1 between tumor tissues and adjacent normal tissues in cases of head and neck cancer. The raw data were processed and normalized using either the GEO2R tool or the R-based limma package, and the statistical significance of the differential expression was evaluated.

### Single-cell RNA-seq analysis

Single-cell transcriptomic sequencing was performed using the 10× Genomics platform. Tumor samples were obtained from two groups: wild-type (WT) mice (*n* = 3) and *Zbp1*^−/−^ mice (*n* = 3), both of which harbored MOC1 cell-derived tongue tumors. Data preprocessing, normalization, and clustering were conducted using Seurat v3.0. To mitigate batch effects across samples, the Harmony algorithm was implemented. Principal component analysis (PCA) and UMAP were used for dimensionality reduction and visualization. Tumor cells were identified using CopyKAT, and differential gene enrichment was analyzed using KEGG and GO databases. GSEA and GSVA were used for gene set enrichment and variation analysis (gene sets from MSigDB v6.2)[28]. Monocle 2 was used for pseudotime trajectory analysis, SingleR for cell type annotation, pySCENIC for transcription factor analysis, and CellPhoneDB for intercellular communication analysis (*p* < 0.05). Comprehensive descriptions of these analyses are available in the Supplementary Materials.

### Immunohistochemistry (IHC)

Tumor tissues were fixed in 4% paraformaldehyde, paraffin-embedded, and sectioned (4 µm). After antigen retrieval with citrate buffer and blocking with 3% H₂O₂ and 10% goat serum, primary antibodies (CCR1, ABclonal, 1:200; CCL7, ABclonal, 1:200; Ki-67, Abcam, 1:200; β-catenin, CST, 1:200; Vimentin, CST, 1:200) were incubated overnight at 4 °C. Secondary antibodies were applied the following day, and DAB staining with hematoxylin counterstaining was performed. H-score analysis was used for quantitative evaluation using ImageJ and the IHC Profiler plugin [29]. H-score incorporates staining intensity (i) and the proportion of positively stained cells (Pi), calculated as:

$${\text{H-score}}\,=(0\times {{\rm{P}}}_{0})+(1\times {{\rm{P}}}_{1})+(2\times {{\rm{P}}}_{2})+(3\times {{\rm{P}}}_{3}),$$

where i = 0 (negative), 1 (weak), 2 (moderate), 3 (strong).

### Immunofluorescence staining

Tissue sections underwent deparaffinization, followed by antigen retrieval and blocking procedures. Subsequently, they were incubated overnight at 4 °C with primary antibodies targeting α-SMA (Proteintech, 1:200) and CCL7 (ABclonal, 1:200). Post-washing, fluorescent secondary antibodies were administered, and the nuclei were counterstained using DAPI. Images were captured utilizing a Nikon confocal microscope. Quantification and analysis of fluorescence signals were conducted with ImageJ software. To evaluate the co-expression of α-SMA (red) and CCL7 (green), the overlapping area of red and green fluorescence signals was quantified and expressed as a ratio relative to the total area of regions positive for red and green fluorescence.

### RNA Extraction and RT-qPCR

Total RNA was extracted using a commercial kit (NCM Biotech, China). Purity was assessed via A260/A280 (1.8–2.2) and A260/A230 ( > 1.7) ratios. Reverse transcription was performed using PrimeScript™ RT Master Mix (Takara, Japan), and qPCR was carried out with SYBR Green Master Mix (MedChemExpress, USA). Primer sequences are listed in Supplementary Table 1. The 2^−ΔΔCt method was used for relative expression analysis.

### Protein extraction and western blotting

Cell lysates were prepared using RIPA buffer, and protein concentrations were determined by BCA assay. Proteins were separated by SDS-PAGE (4–12%) and transferred to PVDF membranes (0.45 µm). Membranes were blocked with 5% non-fat milk for 1 h, incubated with primary antibodies overnight at 4 °C, and then with HRP-conjugated secondary antibodies. Bands were visualized using the Bio-Rad ChemiDoc XRS+ imaging system.

### ZBP1 Gene Silencing

ZBP1 and control sgRNAs (SYNBIO, Suzhou, China) were cloned into the lentiCRISPR v2 vector. Lentivirus was packaged in 293 T cells using Lipofectamine 3000 and used to infect MEFs. Gene silencing efficiency was verified by Western blot. sgRNA sequences are provided in supplementary Table 1.

### Migration and invasion assays

Transwell assays were used to assess cell migration and invasion. MOC-1 cells (4 × 10⁴) were seeded into the upper chamber with serum-free medium; the lower chamber contained CAFs and medium with 10% FBS. For invasion assays, the upper chamber was pre-coated with Matrigel (Corning, USA). After 48 h incubation at 37 °C, non-migrated cells were removed, and migrated cells were fixed with 4% PFA, stained with 0.1% crystal violet, and counted under a microscope.

### EdU cell proliferation assay

The EdU assay was performed using a commercial kit. MOC-1 cells (5 × 10⁴) were seeded in 24-well plates and co-cultured with CAFs for 24 h. EdU (10 μM) was added for 2 h, followed by washing, fixation with 4% PFA, permeabilization with 0.5% Triton X-100, and incubation with the EdU detection reagent for 30 min in the dark. Nuclei were counterstained with Hoechst 33342, and EdU-positive cells were counted under a fluorescence microscope.

### ELISA Assay

Tongue tumors were weighed and homogenized in RIPA buffer containing 10 mM PMSF and protease inhibitors (100 mg/mL). Lysates were sonicated and centrifuged at 12,000 rpm for 10 min at 4 °C. The supernatants were used for measuring CCL7 concentrations in both tumor tissue and cell culture supernatants using a mouse CCL7 ELISA kit (Cusabio, China), following the manufacturer’s instructions.

### Statistical analysis

Statistical analysis was conducted using GraphPad Prism v10 and SPSS 26.0. The Shapiro-Wilk test was used for normality assessment, and Levene’s test for homogeneity of variance. For normally distributed data, Student’s *t*-test or one-way ANOVA (with Tukey’s post hoc test) was applied; otherwise, non-parametric tests (Mann-Whitney U, Kruskal-Wallis) were used. A *p*-value < 0.05 was considered statistically significant.

## Results

### ZBP1 is highly expressed in HNSCC, and its deficiency reduces tumor growth in both orthotopic and chemically induced OSCC mouse models

**Fig. 1:**
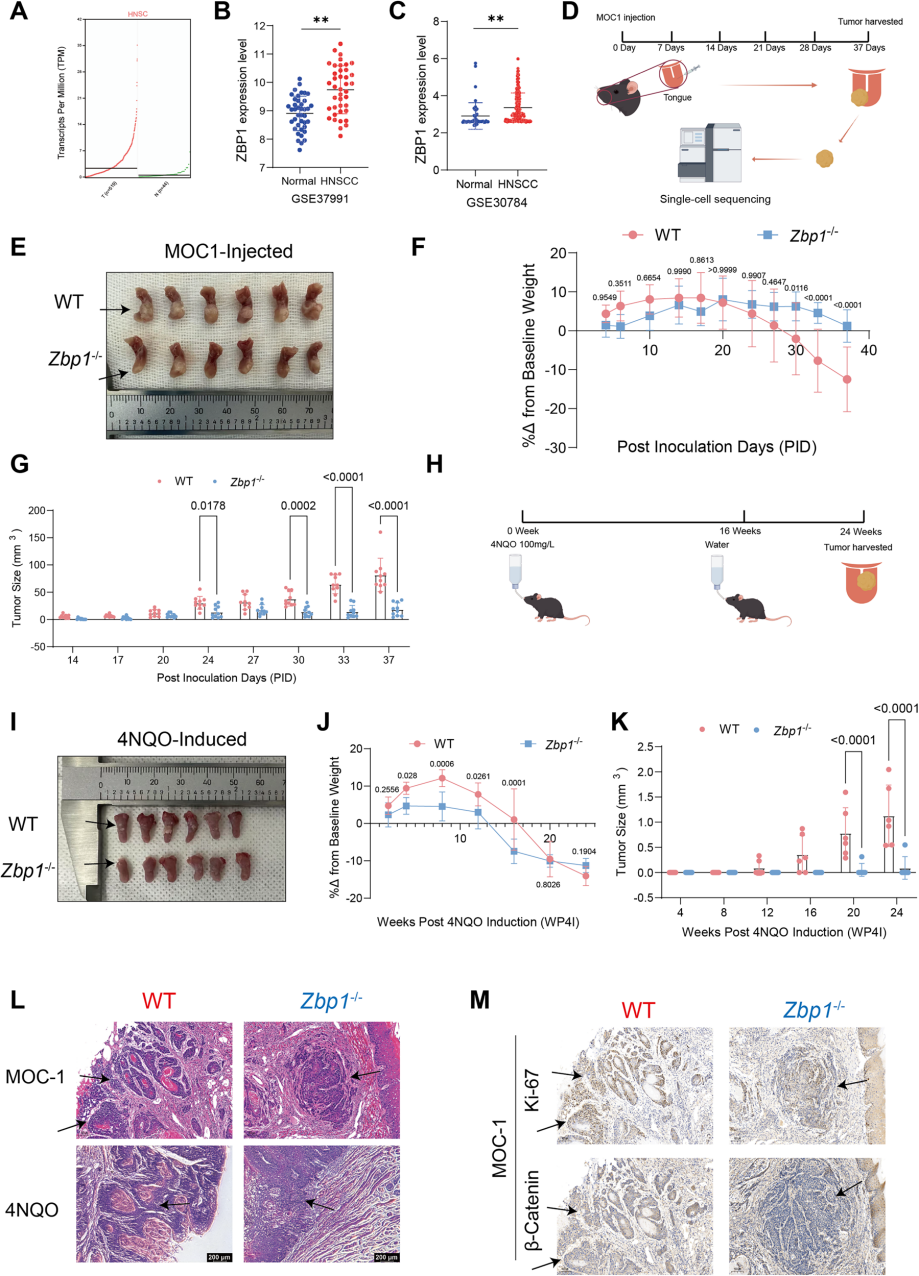
ZBP1 is highly expressed in HNSCC, and its deficiency reduces tumor growth in both orthotopic and chemically induced OSCC mouse models. **A** An analysis of ZBP1 expression in HNSC was conducted utilizing the GEPIA2 platform. **B**, **C** Expression levels of ZBP1 in HNSCC based on GEO datasets. **D** A schematic representation of the single-cell RNA sequencing (scRNA-seq) workflow is presented. Tumors derived from *Zbp1*-deficient and wild-type (WT) mice were dissociated into individual cells, subsequently captured utilizing the 10× Genomics platform, and further processed for library construction and RNA sequencing. **E** The Orthotopic implantation of MOC-1 cells into the tongues of both *Zbp1*^−/−^ and WT mice was performed. **F** Analysis of body weight alterations in WT and *Zbp1*^−/−^ mice bearing tumors following orthotopic implantation of MOC-1 cells. **G** Evaluation of tumor growth trajectories in WT and *Zbp1*^−/−^ mice with orthotopically implanted MOC-1 tumors. **H** Schematic of the 4NQO-induced OSCC model in WT and *Zbp1*^−/−^mice. **I** Presentation of representative gross images depicting OSCC progression induced by 4NQO in WT and *Zbp1*^−/−^ mice. **J** Assessment of body weight fluctuations in tumor-bearing WT and *Zbp1*^−/−^ mice during the 4NQO-induced OSCC model. **K** Analysis of tumor growth dynamics in 4NQO-induced OSCC in WT and *Zbp1*^−/−^ mice. **L** Hematoxylin and eosin (H&E) staining of tumor tissues from both the MOC-1 orthotopic model and the 4NQO-induced model in WT and *Zbp1*^−/−^ mice. **M** Immunohistochemical analysis of Ki-67 and β-catenin expression in tumors derived from MOC-1 orthotopic implantation in WT and *Zbp1*^−/−^mice.

Analysis of the GEPIA2 database using the TCGA–HNSCC cohort revealed significantly upregulated ZBP1 mRNA expression in HNSCC tissues compared to adjacent normal tissues (Fig. 1A). This was validated using independent datasets from the GEO databases (GSE37991 and GSE30784) (Fig. 1B, C). To evaluate the function of ZBP1 in OSCC progression, a dual-model validation system was established: an orthotopic xenograft model generated by transplanting MOC1 cells into the tongues of C57BL/6 mice and a spontaneous OSCC mouse model induced by 4-nitroquinoline-1-oxide (4NQO) administration in drinking water (Fig. 1D, H). Notably, MOC1 transplantation models demonstrated significantly reduced tumor volumes in *Zbp1*^−/−^ mice compared to wild-type (WT) controls (Fig. 1E). Dynamic monitoring data revealed that in the MOC1 model, WT tumors exhibited exponential growth, reaching 80.48 ± 32.21 mm³ by day 37, accompanied by progressive weight loss (12.49 ± 8.31% decrease from baseline). In stark contrast, *Zbp1*^−/−^ mice showed markedly attenuated tumor growth, with final volumes of only 18.51 ± 12.01 mm³, and minimal weight reduction (1.22 ± 4.16%, *p* = 0.32) (Fig. 1F, G). These findings were corroborated using the 4NQO-induced OSCC model (Fig. 1I–K). Consistent with the observations from the MOC1 transplantation models, *Zbp1*^−/−^ mice demonstrated a significantly reduced tumor burden, thereby reinforcing the model-independent inhibitory role of ZBP1 deficiency in the progression of OSCC. While a temporarily accelerated decline in body weight was noted in *Zbp1*^−/−^ mice during the intermediate stages, the overall weight changes between the two groups did not exhibit statistical significance at the study’s conclusion. Histopathological analysis (H&E staining) confirmed the typical OSCC features, including keratin pearl formation and cellular atypia, in both models. In the 4NQO model, *Zbp1*^−/−^ mice predominantly developed mild-to-moderate epithelial dysplasia, whereas WT mice uniformly progressed to invasive carcinoma (Fig. 1L). Immunohistochemical analysis demonstrated an increased expression of the proliferation marker Ki67, along with an upregulation of β-catenin, a pivotal molecule in the Wnt signaling pathway, in wild-type tumors derived from the MOC1 model (Fig. 1M). Collectively, these results demonstrated that ZBP1 deficiency effectively suppressed the malignant progression of OSCC.

### Analysis of tumor cell heterogeneity between *Zbp1*^−/−^ and WT groups

**Fig. 2:**
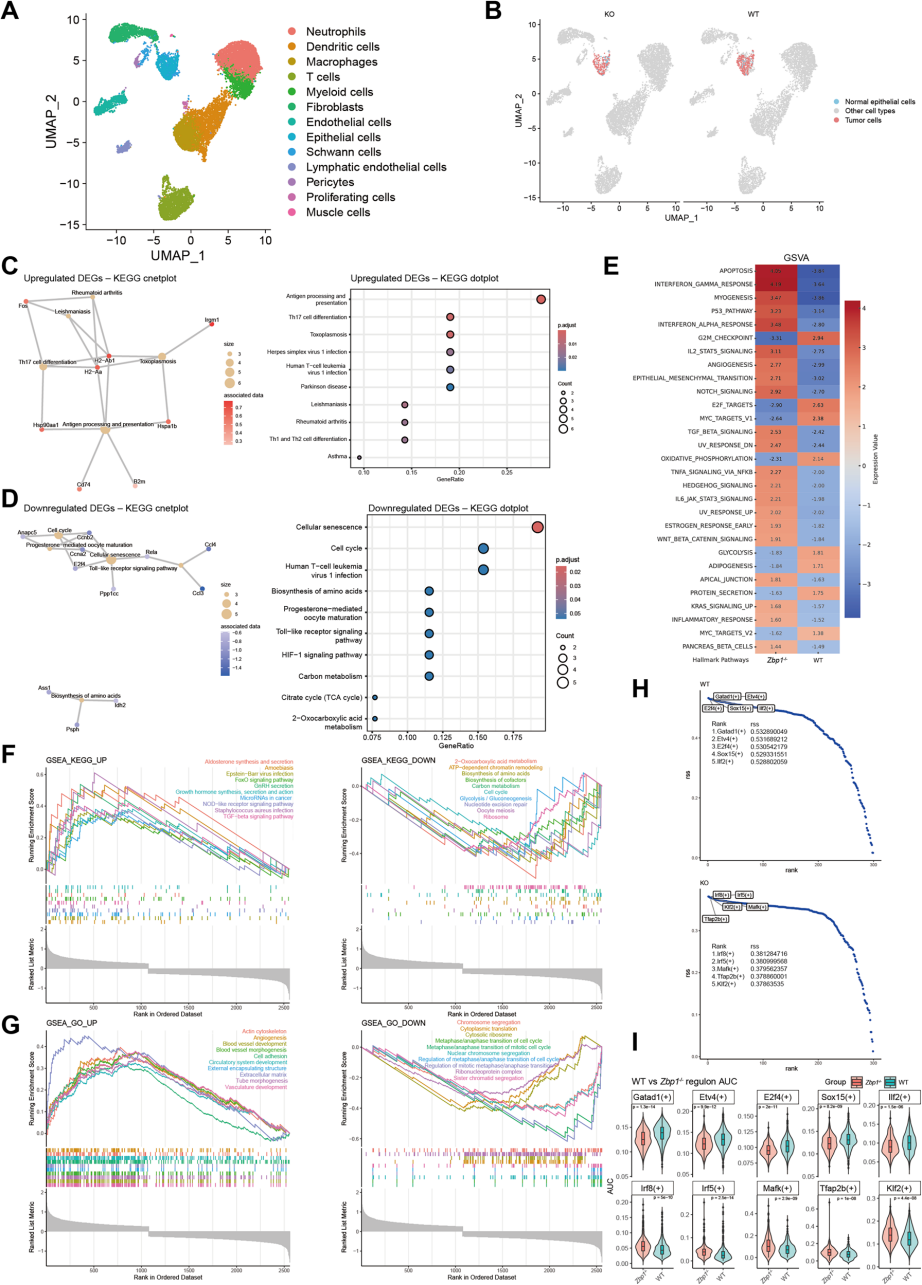
Analysis of tumor cell heterogeneity between *Zbp1*^−/−^ and WT groups. **A** Thirteen major cell types were identified using the Seurat R package and visualized by UMAP. **B** Tumor cells in *Zbp1*^*−/−*^ and WT groups were identified using CopyKAT. **C** The KEGG enrichment analysis was conducted on the upregulated DEGs in tumor cells from the *Zbp1*^−/−^ group. The left panel presents a KEGG cnetplot illustrating the associations between enriched pathways (category nodes) and their corresponding DEGs (gene nodes). The size of each node reflects the number of DEGs involved in the respective pathway, while the node color denotes the adjusted *p*-value. The right panel displays a KEGG dotplot of the same dataset, where the x-axis represents the GeneRatio. The size of each bubble indicates the number of DEGs, and the bubble color signifies the adjusted *p*-value. **D** The KEGG enrichment analysis of downregulated DEGs in tumor cells from the *Zbp1*^−/−^ group is presented. The left panel displays a KEGG cnetplot, maintaining consistent node size and color coding as depicted in panel (**C**). The right panel features a KEGG dotplot, preserving the same axis, size, and color coding as in panel (**C**). KEGG enrichment analyses were conducted independently for upregulated and downregulated differentially expressed genes (DEGs), with pathways deemed significant at at adjusted *p* < 0.05. The GeneRatio represents the proportion of DEGs associated with each pathway. Given the variation in the total number of DEGs between the two groups, the GeneRatio and node sizes are comparable solely within each respective group. **E** GSVA enrichment analysis of Hallmark pathways for differentially expressed genes between tumor cells in *Zbp1*^*−/−*^ and WT groups. **F**, **G** GSEA analyses reveal differences in KEGG and GO pathway enrichment between tumor cells from *Zbp1*^*−/−*^ and WT mice. Pathways were considered significant if they exhibited an absolute normalized enrichment score (|NES | ) greater than 1, a nominal *p*-value less than 0.05, and an adjusted *p*-value (false discovery rate, FDR) below 0.25. The top 10 upregulated pathways (depicted in the left panel) and the top 10 downregulated pathways (depicted in the right panel) were selected based on their NES values. The X-axis represents the rank of genes within the ordered dataset, determined by log₂ fold change. The Y-axis in the upper panel illustrates the Running Enrichment Score (ES) for each pathway, while the Y-axis in the lower panel displays the ranked list metric, specifically the log₂ fold change, for each gene. **H** Regulatory specificity scores (RSS) of the top five transcription factors differ between *Zbp1*^*−/−*^ and WT groups. Regulons were ranked by rss within each group (y-axis: rss; x-axis: rank). Filtering thresholds (zThreshold=0.3, thr = 0.01) were applied, and top-ranked regulons are annotated. **I** Comparison of regulon activities between WT and *Zbp1*^−/−^ groups. Each violin represents the density distribution of single-cell AUC values, with an embedded boxplot indicating the median and interquartile range. Individual dots represent outlier cells outside 1.5 × IQR. Statistical significance between WT and *Zbp1*^−/−^ groups was assessed using the Wilcoxon rank-sum test; corresponding *p*-values are shown above each panel.

To elucidate the TME-associated mechanisms underlying ZBP1-mediated regulation of OSCC growth, a dual-model parallel experimental design was implemented wherein equal numbers of MOC1 cells were orthotopically inoculated into the tongues of WT and *Zbp1*^−/−^mice. Tumor tissues were harvested at a standardized endpoint (day 35 post-inoculation; *n* = 3/group) for single-cell transcriptome sequencing using a 10× Genomics platform. Following multiparameter quality control (genes > 500; mitochondrial gene fraction < 20%), 13 572 high-quality single cells were retained. Through UMAP dimensionality reduction clustering and SingleR annotation, cross-referenced with published HNSCC atlases, cells were classified into 13 functional subsets: neutrophils (S100a9^+^/S100a8^+^) [30], dendritic cells (H2-Ab1^+^/CD74^+^) [31], macrophages (CD68^+^/ADGRE1^+^) [32], T cells (CD3G^+^/TRAC^+^) [33], myeloid progenitors (CEBPB^+^/CSF3R^+^) [34], cancer-associated fibroblasts (DCN^+^/COL1A2^+^) [35], vascular endothelial cells (PECAM1^+^/ESAM^+^) [36], epithelial cells (EPCAM^+^/KRT5^+^) [37], Schwann cells (SOX10^+^) [38], lymphatic endothelial cells (PROX1^+^) [39], pericytes (RGS5^+^)[40], proliferating cells (MKI67^+^/STMN1^+^) [41], and myogenic cells (ACTA1^+^) [42] (Fig. 2A, Supplemental Fig. 2A). The proportions of functional cell subpopulations are shown in Supplemental Fig. 2B. Malignant cell identification using CopyKAT revealed the exclusive origin of aneuploid cells from the epithelial cluster (purity > 0.85), with genomic instability signatures closely mirroring those of clinical OSCC samples (Fig. 2B).

Tumor cells within the epithelial cluster were identified using CopyKAT. Differentially expressed genes (DEGs) between tumor cells from WT and *Zbp1*^−/−^ mice were analyzed. KEGG enrichment analysis revealed that immune-related pathways, such as antigen processing and presentation, were upregulated in *Zbp1*^−/−^ tumor cells (Fig. 2C). In contrast, metabolism- and proliferation-associated pathways, including cellular senescence, cell cycle, Toll-like receptor signaling, and HIF-1 signaling pathways, were downregulated (Fig. 2D). Gene Set Variation Analysis (GSVA) revealed marked transcriptomic reprogramming in *Zbp1*^−/−^ tumor cells. Compared to WT controls, key proliferation-related pathways, including the G2/M checkpoint, E2F targets, and MYC targets, were significantly downregulated in *Zbp1*^−/−^ tumor cells, indicating impaired cell cycle progression and proliferative capacity. The concurrent downregulation of metabolic pathways (e.g., oxidative phosphorylation and glycolysis) reflected reduced metabolic activity, suggesting a hypometabolic state. Notably, multiple stress-responsive and tumor-suppressive pathways were upregulated in *Zbp1*^−/−^ tumor cells, including p53 signaling and apoptosis, implying the activation of programmed cell death. Immune-related pathways—interferon (IFN)-α/γ response, IL-2–STAT5 signaling, IL-6–JAK–STAT3 signaling, and TNF-α–NF-κB signaling—were also elevated, indicating potential immune stress or surveillance states. Simultaneously, significant enrichment was observed in TGF-β signaling, Notch signaling, WNT/β-catenin signaling pathways. The upregulation of the apical junction pathway indicates a remodeling of cell adhesion structures and, in conjunction with the heightened expression of angiogenesis-related genes, likely represents compensatory adaptations to the stress within the tumor microenvironment induced by the loss of ZBP1. Nonetheless, despite the activation of these potentially pro-tumorigenic pathways, tumors in *Zbp1*^*−/−*^ mice were significantly smaller, suggesting that these responses may be inadequate to counteract the overall inhibitory effect of ZBP1 deficiency on tumor progression (Fig. 2E).

Gene Set Enrichment Analysis (GSEA) demonstrated a significant downregulation of several pathways associated with cell proliferation and metabolism in *Zbp1−/−* tumor cells. These findings were further substantiated by KEGG analysis, which revealed a significant upregulation of pathways associated with stress adaptation, immune regulation, and signal transduction, particularly the TGF-β signaling pathway, FoxO signaling pathway, NOD-like receptor signaling pathway, and microRNA pathways in cancer (Fig. 2F, left panel). This finding suggests that tumor cells activate tumor-suppressive, stress-adaptive, and immune-regulatory mechanisms following the loss of ZBP1. In contrast to the upregulated pathways, KEGG analysis pronounced downregulation in pathways related to the cell cycle, glycolysis/gluconeogenesis, carbon metabolism, and ribosomal function (refer to Fig. 2F, right panel). These results suggest that the absence of ZBP1 markedly impairs tumor cell proliferation and metabolic capacity, thereby limiting rapid tumor growth. Concurrently, GO enrichment analysis indicated an upregulation of processes related to angiogenesis, blood vessel development, cell adhesion, and tube morphogenesis (Fig. 2G, left panel), which reflects the adaptive remodeling of tumor cells in response to changes in the tumor microenvironment (TME). Additionally, Gene Ontology (GO) analysis identified the suppression of various biological processes, including chromosome segregation, metaphase/anaphase transition, cytoplasmic translation, and ribosomal function (Fig. 2G, right panel).

Utilizing single-cell transcriptomic data as a foundation, SCENIC analysis was conducted to investigate the impact of ZBP1 deletion on transcriptional regulatory networks within tumor cells. The absence of ZBP1 resulted in a reconfiguration of transcription factor (TF) regulatory circuits in these cells. In *Zbp1*^*−/−*^ tumors, transcription factors such as IRF8, IRF5, and KLF2 were identified as top regulons based on their regulon specificity scores (rss), indicating increased activity. These transcription factors are recognized for their collaborative role in activating immune surveillance and programmed cell death pathways by regulating interferon response elements and genes associated with apoptosis [43–45]. Simultaneously, elevated activities of MAFK and TFAP2B were linked to cell cycle suppression and heightened G1 phase arrest [46, 47]. Conversely, WT tumor cells showed leading proliferation-promoting TFs (E2F4 and ETV4), aligning with rapid cell cycle advancement and glycolytic reprogramming [48–50] (Fig. 2H). Abnormal activation of GATAD1 and ILF2 was associated with invasive tumor characteristics [51, 52]. Importantly, rss values are are designed for ranking within groups rather than for direct comparisons across groups, as they indicate relative specificity within each population. To further assess whether the top five-ranked regulons identified through RSS analysis exhibited differential activities between WT and *Zbp1*^*−/−*^ tumor cells, we conducted direct AUC comparisons at the single-cell level. Violin plots demonstrated significantly elevated activities of IRF5, IRF8, KLF2, TFAP2B, and MAFK in *Zbp1*^*−/−*^ tumor cells, while E2F4 and ETV4 were preferentially activated in WT tumors (Fig. 2I). Collectively, these findings suggest that ZBP1 deficiency is linked to a regulatory shift from proliferation–invasion pathways towards immune–apoptotic regulons, thereby offering a plausible mechanistic explanation for its tumor-suppressive function.

### Pseudotime trajectory analysis of tumor cells in *Zbp1*^*−/−*^ and WT groups

**Fig. 3:**
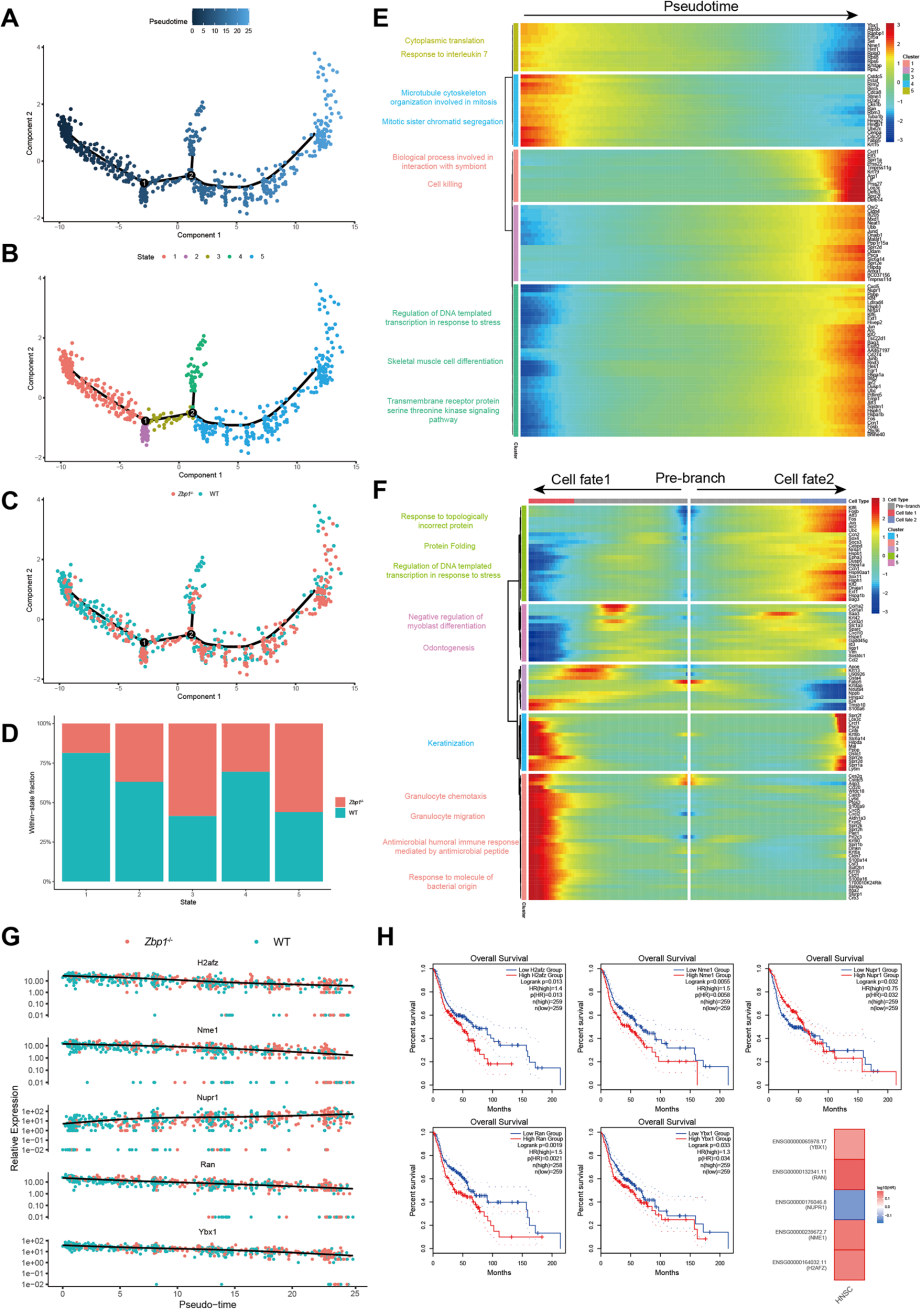
Pseudotime trajectory analysis of tumor cells in *Zbp1*^*−/−*^ and WT groups. **A**–**C** Pseudotime analysis of tumor cells using Monocle2. Each dot on the graph denotes an individual cell, systematically arranged according to the inferred developmental trajectory. The x-axis and y-axis, labeled as Component 1 and Component 2, respectively, represent the two principal dimensions obtained through DDRTree-based dimensionality reduction. **A** Cells ordered along pseudotime. **B** Cells grouped into distinct ‘states’, which represent transcriptionally similar cell clusters along the trajectory. **C** Distribution of WT and *Zbp1*^−/−^ tumor cells along the trajectory. **D** Proportion of tumor cells from WT and Zbp1−/− mice within each state identified in Fig. 3B. **E** Heatmap of functional enrichment for the top 100 differentially expressed genes. **F** Heatmap showing differentially expressed genes along distinct branches and GO Biological Process (GO BP) enrichment across clusters. **G** Dynamic expression patterns of representative differentially expressed genes that determine cell fate in *Zbp1*^*−/−*^ and WT tumor cells. **H** Survival analysis of genes from (**G**) using the GEPIA2 database.

To enhance our comprehension of tumor cell evolution, we employed the Monocle2 algorithm to delineate the developmental trajectory of tumor cells. Our pseudotime analysis demonstrated that WT and *Zbp1*^−/−^ tumor cells are positioned distinctly along the inferred trajectory, with WT tumor cells predominantly occupying early developmental states, while *Zbp1*^−/−^ tumor cells are more prevalent at terminal states (Fig. 3A–D, Supplemental Fig. 3A). To quantitatively substantiate this observation, we conducted a comparative analysis of the distribution of cells across pseudotime states between the two groups. A total of 431 wild-type (WT) and 275 *Zbp*1^−/−^ tumor cells were examined. In the WT cohort, 48.03% of cells were situated in state 1 and 29.47% in state 5. Conversely, in the *Zbp*1^−/−^ cohort, only 17.09% of cells were found in state 1, whereas 58.91% were in state 5 (Supplemental Fig. 3A). Further analysis within each state revealed that WT cells constituted 81.50% of state 1, while *Zbp*1^−/−^ cells comprised 56.05% of state 5 (Fig. 3D), thereby reinforcing the notion of preferential enrichment of *Zbp*1^−/−^ cells in terminal pseudotime states. This observation indicates a substantial divergence in transcriptional progression between the two groups, implying potential differences in their differentiation dynamics. We identified the top 100 genes exhibiting the most significant dynamic changes along the pseudotime trajectory in malignant epithelial cells derived from both WT and *Zbp1*^−/−^ groups. These genes were clustered into five representative modules based on their temporal expression patterns. Subsequent Gene Ontology (GO) enrichment analysis revealed distinct biological programs associated with different pseudotime stages (Fig. 3E). Early pseudotime clusters, enriched in WT group tumor cells, were predominantly associated with cytoplasmic translation and mitotic processes, reflecting active protein synthesis and proliferative states (Fig. 3E). In contrast, late pseudotime clusters, enriched in *Zbp1*^−/−^ tumor cells, were linked to stress-responsive transcriptional regulation, receptor signaling pathways, and differentiation-related processes, suggesting a shift toward adaptive and less proliferative cellular states (Fig. 3E). Following this, we conducted BEAM analysis on branch point 2, which is a pivotal node influencing cell fate decisions. In this analysis, the pre-branch corresponds to state 1 and state 3, while the two trajectories diverging from branch point 2 are defined as cell fate 1 (state 4) and cell fate 2 (state 5). Notably, state 4 contained a higher proportion of WT-derived tumor cells, whereas state 5 was predominantly composed of *Zbp1*^−/−^ derived tumor cells (Fig. 3D). The results showed that genes highly expressed in the fate 1 trajectory were enriched in pathways such as Granulocyte chemotaxis, Granulocyte migration, Antimicrobial humoral immune response mediated by antimicrobial peptide, and Response to molecule of bacterial origin (Fig. 3F). In contrast, genes upregulated in the fate 2 trajectory were mainly enriched in Response to topologically incorrect protein, Protein folding, and Regulation of DNA-templated transcription in response to stress (Fig. 3F). These findings suggest that WT tumor cells preferentially follow an immune and inflammation-associated trajectory, potentially promoting tumor progression, whereas *Zbp1*^−/−^ tumor cells are biased towards stress-adaptive and protein homeostasis programs, which may restrict tumor growth. The analysis of critical regulatory networks identified five prognostic molecular markers within the core trajectory gene cluster. Interestingly, *Zbp1*−/− tumor cells showed lower expression of H2afz, Nme1, Ran, and Ybx1, but higher expression of Nupr1 compared to WT cells (Fig. 3G). An overall survival analysis, conducted utilizing the GEPIA2 web server with the HNSC dataset, indicated that elevated expression levels of H2AFZ, NME1, RAN, and YBX1 are correlated with poorer survival outcomes, whereas the activation of NUPR1 is associated with a more favorable prognosis (Fig. 3H).

### Single-cell interaction network analysis reveals ZBP1-mediated CAF-tumor cell communication via the CCL7–CCR1 axis in OSCC

**Fig. 4:**
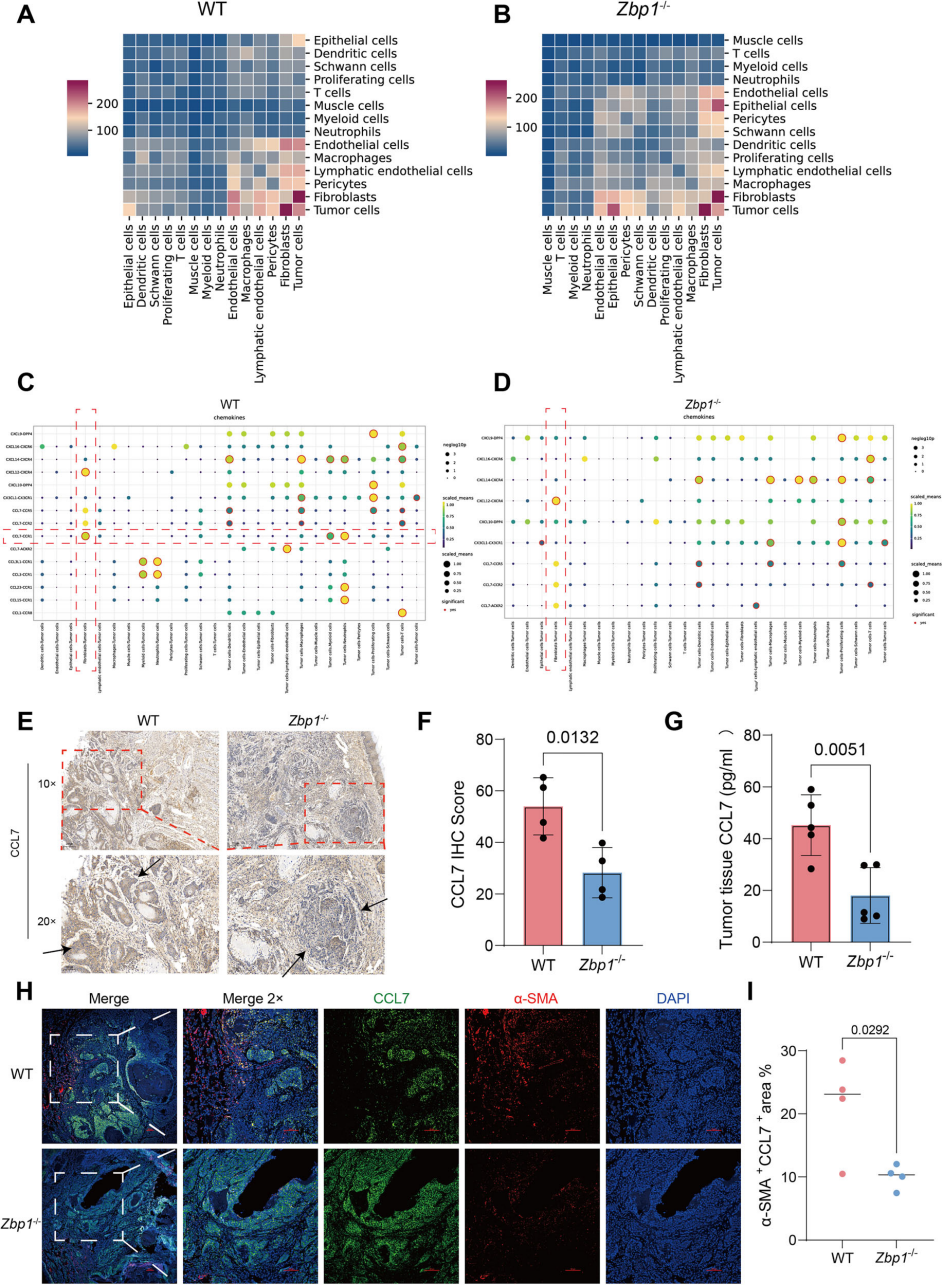
Single-cell interaction network analysis reveals ZBP1-mediated CAF-tumor cell communication via the CCL7–CCR1 axis in OSCC. **A** Heatmap showing cell–cell interactions in the WT group as analyzed by CellPhoneDB. **B** Heatmap showing cell–cell interactions in the *Zbp1*^−/−^ group as analyzed by CellPhoneDB. **C** Dot plot illustrating chemokine-related ligand–receptor interactions in the WT group. **D** Dot plot illustrating chemokine-related ligand–receptor interactions in the *Zbp1*^*−/−*^ group. **E** Immunohistochemistry (IHC) showing CCL7 expression in tumor tissues from WT and *Zbp1*^−/−^ mice. **F** Quantification of CCL7 IHC scores. **G** ELISA analysis of CCL7 protein levels in tumor tissues from WT and *Zbp1*^*−/−*^ mice. **H** Immunofluorescence analysis of CCL7 expression in CAFs within tumor tissues of WT and *Zbp1*^*−/−*^ mice. **I** Quantitative analysis of CCL7⁺/α-SMA⁺ co-localized regions in tumor sections.

Panoramic cell-cell interaction analysis using CellPhoneDB demonstrated that tumor cell-CAF communication networks dominated the OSCC microenvironment (Fig. 4A, B). KEGG enrichment analysis of differentially expressed genes in CAFs between the two groups revealed a significant downregulation of chemokine signaling pathways in CAFs from the *Zbp1*^−/−^ group (Supplemental Fig. 4A, B). The analysis of chemokine signaling pathways demonstrated that a deficiency in ZBP1 selectively inhibited the CCL7–CCR1 signaling axis between CAFs and tumor cells. This inhibition was evident as a marked reduction in signaling intensity within the spatial transcriptomic interaction heatmaps (Fig. 4C, D). Additionally, the unfiltered chemokine dot plot, which displays all pairings, can be found in Supplemental Fig. 5A and Supplemental Fig. 6A. Correspondingly, a single-cell dot plot analysis indicates that the expression of *Ccr1* in tumor cells is significantly reduced in *Zbp1*^*−/−*^ mice compared to wild-type (WT) counterparts (Supplemental Fig. 6B). Immunohistochemical co-staining confirmed the systemic downregulation of CCL7 expression in the tumor parenchymal and stromal regions of *Zbp1*^−/−^ mice (Fig. 4E, F). An enzyme-linked immunosorbent assay (ELISA) further demonstrated substantially reduced CCL7 secretion in tumor tissue homogenates (Fig. 4G). To spatially resolve CCL7 cellular sources, α-SMA/CCL7 dual immunofluorescence was performed. Wild-type tumors exhibited abundant CCL7^+^ CAFs clustered at the invasive front, with significantly higher fluorescence co-localization intensity than *Zbp1*^−/−^ counterparts (Fig. 4H, I). This spatial distribution pattern aligns with ligand–receptor interaction models, indicating that ZBP1 likely modulates stromal–tumor cascade communication by regulating CAF chemokine secretion.

### MOC1-induced CAFs promote invasion, migration, and proliferation of MOC1 cells

**Fig. 5:**
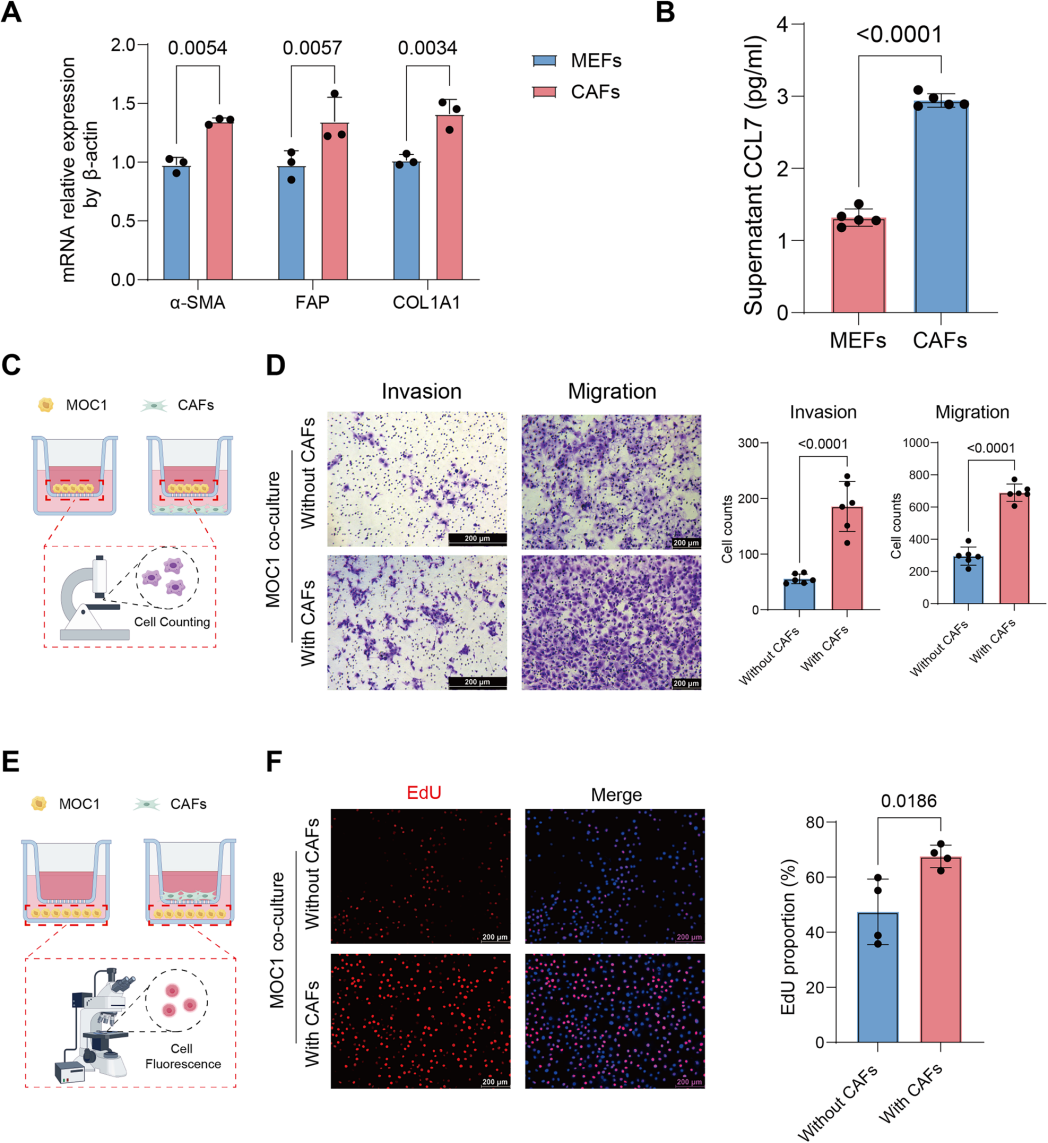
MOC1-induced CAFs promote invasion, migration, and proliferation of MOC1 cells. **A** qRT-PCR analysis confirming the induction of MEFs into CAFs after exposure to MOC1-conditioned medium. **B** ELISA quantification of CCL7 levels in the supernatants of MEFs and CAFs. **C** Schematic diagram of the Transwell assay used to assess MOC1 cell migration and invasion under co-culture with CAFs. **D** Migration and invasion of MOC1 cells after 48 h in the control group versus co-culture with CAFs. **E** Schematic diagram of the EdU assay used to assess MOC1 cell proliferation under co-culture with CAFs. **F** MOC1 cell proliferation after 24 h in the control group versus co-culture with CAFs, as assessed by EdU assay.

To model CAFs in vitro, a co-culture system was employed in which mouse embryonic fibroblasts (MEFs) were exposed to tumor-conditioned medium (TCM) from MOC1 cells, inducing a transformation toward a CAF-like phenotype. This method effectively recapitulates paracrine-mediated fibroblast activation in the TME, while avoiding interference from direct cell contact [53]. Following 72 h of co-culture, MEFs acquired characteristic CAF markers with significant upregulation of α-SMA (ACTA2), FAP, and COL1A1 (Fig. 5A). ELISA results further confirmed the elevated CCL7 secretion in CAF culture supernatants compared to uninduced MEFs (Fig. 5B).

A Transwell co-culture system (Fig. 5C) demonstrated that MOC1 cells co-cultured with CAFs exhibited significantly enhanced invasion and migration capabilities (Fig. 5D). Furthermore, EdU assays revealed increased proliferative activity through higher EdU^+^ cell ratios in the co-cultured MOC1 cells (Fig. 5E, F). Collectively, these findings indicated that CAFs significantly potentiated the invasive, migratory, and proliferative capabilities of MOC1 tumor cells.

### ZBP1 deficiency impairs CAF-mediated CCL7 secretion and attenuates tumor cell malignancy via the CCL7–CCR1 axis

**Fig. 6:**
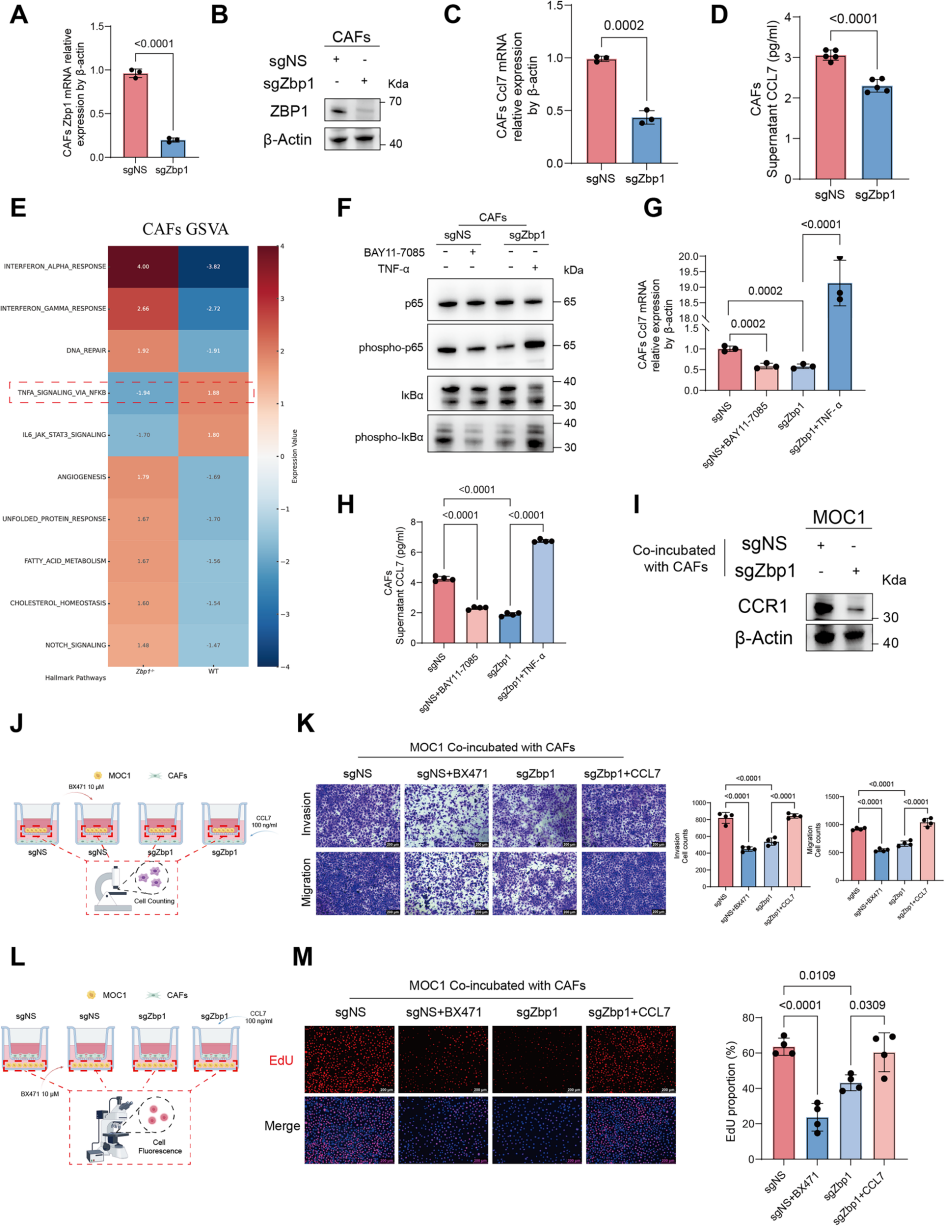
ZBP1 deficiency impairs CAF-mediated CCL7 secretion and attenuates tumor cell malignancy via the CCL7–CCR1 axis. **A** qRT-PCR validation of ZBP1 mRNA deficiency in CAFs. **B** Western blot analysis was conducted to confirm the deficiency of ZBP1 in CAFs. **C** qRT-PCR analysis of CCL7 mRNA expression in CAFs under control conditions and following ZBP1 deficiency. **D** ELISA was utilized to measure the levels of CCL7 in the supernatant of CAFs before and after ZBP1 deficiency. **E** GSVA heatmap was generated to illustrate the activities of hallmark pathway in CAFs from WT and *Zbp1*^−/−^ groups. **F** Western blot analysis was performed to evaluate p65/IκBα signaling to assess NF-κB pathway activity in CAFs. Samples include sgNS controls, sgNS treated with BAY11-7085 (NF-κB inhibitor), sgZbp1, and sgZbp1 treated with TNF-α (an NF-κB activator). **G** qRT-PCR analysis was conducted to assess Ccl7 mRNA expression in CAFs. **H** ELISA analysis was performed to determine the levels of secreted CCL7 levels in CAF supernatants. **I** Western blot analysis was conducted to assess CCR1 expression in MOC1 cells co-cultured with differently treated CAFs (sgNS or sgZbp1). **J** A schematic diagram illustrates the Transwell assay employed to evaluate the impact of various co-culture conditions on the migration and invasion of MOC1 cells. **K** The migration and invasion capabilities of MOC1 cells were analyzed after 48 h under different co-culture conditions, accompanied by statistical analysis. **L** A schematic diagram depicts the EdU assay used to evaluate MOC1 cell proliferation under varying co-culture conditions. **M** The proliferation of MOC1 cells was assessed after 24 h under different co-culture conditions, with accompanying statistical analysis.

To delineate ZBP1’s regulatory role in CAFs, CRISPR-Cas9 technology was employed to construct lentiviral vectors targeting ZBP1. Efficient gene silencing in CAF derivatives was confirmed by qRT-PCR and western blotting (Fig. 6A–C). ELISA revealed significantly reduced CCL7 secretion in conditioned media from ZBP1-deficient CAFs compared to controls (Fig. 6D). GSVA of CAFs indicated a downregulation of the NF-κB signaling pathway in the *Zbp1*^*−/−*^ group, which may consequently contribute to a reduction in CCL7 expression (Fig. 6E). Cells were treated independently with NF-κB inhibitors and agonists (Fig. 6F). The results demonstrated that inhibition of NF-κB led to a significant downregulation of Ccl7 expression, whereas activation of NF-κB resulted in an upregulation of Ccl7 expression (Fig. 6G, H). Interestingly, analysis of single-cell sequencing data revealed that only the quantity of myCAFs exhibited significant differences between the WT and KO groups, whereas no significant differences were observed in other cell types. Additionally, CCL7 expression was predominantly localized to iCAFs. Further investigation demonstrated that the expression levels of CCL7 in CAFs were significantly elevated in the WT group compared to the KO group (Supplemental Fig. 7A–D).

To investigate whether ZBP1 modulates CCR1 expression in tumor cells via CCL7 produced by CAFs, we employed a co-culture system. The Western blot results showed that co-culture of MOC1 cells with ZBP1-deficient CAFs led to a significant downregulation of CCR1 expression (Fig. 6I). Furthermore, Western blot analysis demonstrated that co-culture of MOC1 cells with ZBP1-deficient CAFs suppressed multiple downstream signaling pathways, including MAPK (ERK1/2), NF-κB (p65, IκBα), and AKT, compared with co-culture with control CAFs. In contrast, STAT3 activation remained largely unchanged. These findings suggest that the attenuation of MAPK, NF-κB, and AKT signaling may underlie the altered biological behaviors of MOC1 cells (Supplemental Fig. 8A, B). To further investigate the mediating role of the CCL7–CCR1 axis, co-culture systems underwent pharmacological interventions (Fig. 6J, L). Functional assays revealed that the ablation of ZBP1 significantly diminished the capacity of CAFs to enhance the invasion, migration, and proliferation of co-cultured MOC1 cells (Fig. 6K, M). The CCR1 inhibitor BX471 (10 μM) effectively inhibited CCR1 signaling in MOC1 cells, while the administration of recombinant murine CCL7 (100 ng/ml) restored the phenotypic characteristics in ZBP1-deficient groups. Transwell assays revealed that BX471 treatment suppressed the invasion and migration of control MOC1 cells. Conversely, exogenous CCL7 effectively reversed the tumor-suppressive effects of ZBP1-deficient CAFs, restoring malignant phenotypes, i.e., invasion (Fig. 6K), migration (Fig. 6K), and proliferation (Fig. 6M). Taken together, these data demonstrated that ZBP1 enhanced tumor cell invasiveness and metastatic potential by regulating CAF-secreted CCL7 to activate CCR1-dependent signaling pathways.

### In vivo validation of the critical role of ZBP1 in OSCC pathogenesis via the CCL7–CCR1 axis

**Fig. 7:**
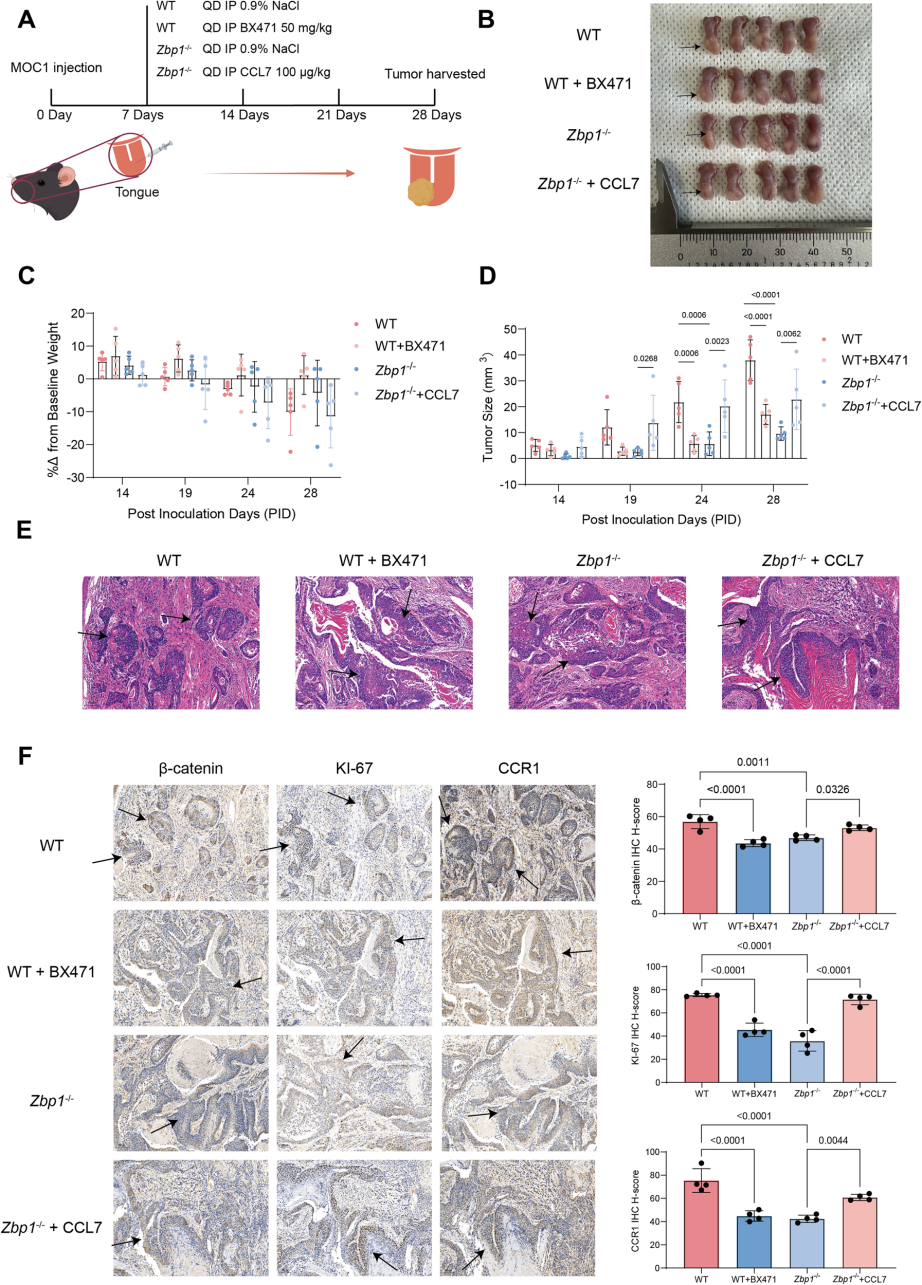
In vivo validation of the critical role of ZBP1 in OSCC pathogenesis via the CCL7–CCR1 axis. **A** Schematic illustration of the experimental design showing orthotopic implantation of MOC1 cells in WT and *Zbp1*^−/−^ mice, along with intraperitoneal injections of BX471 and recombinant CCL7. **B** Tumor formation in four groups: WT, WT + BX471, *Zbp1*^−/−^, and *Zbp1*^−/−^+CCL7. **C** Body weight changes during MOC1 tumor development across different groups. **D** Tumor growth curves during MOC1 tumor progression in the different treatment groups. **E** Hematoxylin and eosin (H&E) staining of tumor tissues from each group. **F** Immunohistochemical analysis and quantification of Ki-67, β-catenin, and CCR1 expression in tumor tissues from the respective groups.

To verify ZBP1’s regulatory function through the CCL7–CCR1 signaling axis in vivo, an orthotopic OSCC model was established by inoculating MOC1 cells into the tongues of WT and *Zbp1*^−/−^ mice. Interventions commenced on day 7 post inoculation (Fig. 7A). WT mice were administered the CCR1-specific inhibitor BX471 (50 mg/kg, i.p., four times daily), while *Zbp1*^−/−^ mice received exogenous recombinant CCL7 protein (100 μg/kg, i.p., four times daily) for three weeks. BX471 treatment significantly reduced tumor volume in WT mice, whereas recombinant CCL7 supplementation markedly increased tumors in *Zbp1*^−/−^ mice (Fig. 7B, D). At the endpoint (day 28), untreated WT mice exhibited tumor volumes of 37.96 ± 7.88 mm³ with 10.10 ± 7.08% body weight loss (Fig. 7B, C). In contrast, BX471-treated WT mice exhibited reduced tumors (17.06 ± 3.87 mm³; *p* < 0.05) and 1.16 ± 5.88% weight gain. Meanwhile, untreated *Zbp1*^−/−^ mice developed smaller tumors (9.65 ± 2.60 mm³) with moderate weight loss (-4.30 ± 9.99%) whereas CCL7-supplemented *Zbp1*^−/−^ mice contained enlarged tumors (22.90 ± 11.65 mm³; *p* < 0.05) and experienced accelerated weight loss (-11.47 ± 9.53%;).Histopathological examination utilizing H&E staining demonstrated that BX471 effectively inhibited tumor progression in wild-type (WT) mice, as illustrated in Fig.7D-E. This was evidenced by the downregulation of Ki-67, CCR1, and invasion-associated β-catenin. Conversely, the administration of CCL7 counteracted these suppressive effects in *Zbp1*^−/−^ tumors (Fig. 7F). Cumulatively, these results demonstrated that ZBP1 deficiency attenuated OSCC progression by downregulating the CCL7–CCR1 axis, while exogenous CCL7 restored oncogenic signaling to reverse the *Zbp1*^−/−^ phenotype.

## Discussion

This study identified the pro-tumorigenic role of ZBP1 in OSCC and elucidated its molecular mechanism in enhancing tumor cell proliferation, migration, and invasion by regulating the CCL7–CCR1 chemokine axis between CAFs and tumor cells. Utilizing a ZBP1-knockout mouse model coupled with single-cell RNA sequencing analysis, ZBP1 deficiency was found to significantly suppress OSCC initiation and progression, underscoring ZBP1’s critical role in modulating the TME to facilitate cancer progression.

Notably, significantly reduced OSCC volume was detected in *Zbp1*^−/−^ mice. Similarly, Karki et al. demonstrated that myeloid-specific ADAR1 deletion in Adar1^fl/fl^ LysMcre mice upregulates ZBP1 expression, activating the RIPK3-mediated PANoptosome complex, a key driver of PANoptosis, to induce pyroptosis, necroptosis, and apoptosis, significantly suppressing colorectal tumor growth. This effect was reversed upon ZBP1 or Zα2 domain knockout, confirming ZBP1’s critical role [54]. Additionally, in B16 melanoma models, combined IFN-γ and KPT-330 treatment induced ZBP1-dependent PANoptosis, effectively inhibiting tumor progression [54]. However, Baik et al. reported that ZBP1 deficiency blocked necroptosis under glucose deprivation and significantly suppressed metastasis in breast cancer models, suggesting context-dependent pro-tumorigenic effects through cell death and inflammation promotion [55]. Collectively, these findings suggest a potential tumor-type- or TME-dependent functional divergence of ZBP1. Evidence indicates that Squamous cell carcinomas (SCC) exhibit high tissue and molecular heterogeneity. In OSCC, this heterogeneity manifests as diverse tumor cell differentiation states, heterogeneous gene expression profiles, and complex immune microenvironments [56].

To investigate the tumor immune microenvironment, scRNA-seq analysis in the current study revealed that ZBP1 deficiency significantly downregulated multiple metabolism- and proliferation-related pathways in tumor cells (e.g., E2F targets, MYC targets, glycolysis, and cell cycle pathways), while upregulating stress response, immune activation, and cell death-associated pathways (including p53 signaling, TNF-α/NF-κB signaling, and interferon responses). These findings suggest that ZBP1 modulates tumor cell fate by regulating TME stress. Further cell–cell interaction analysis demonstrated that tumor cells exhibited the closest proximity to CAFs within the OSCC immune microenvironment. CAFs are pivotal pro-tumorigenic components within the TME across multiple cancer types, with documented functions spanning tumor initiation, progression, immune evasion, and therapeutic resistance.

CAFs remodel the ECM and enhance tumor cell invasiveness by secreting cytokines (e.g., TGF-β, IL-6) and chemokines (e.g., CCL2, CXCL12), promoting angiogenesis and inducing EMT. In breast cancer, FOSL2-expressing CAFs mediate VEGF-independent angiogenesis through the transcriptional activation of WNT5A, revealing novel anti-angiogenic therapeutic strategies targeting the tumor stroma [57]. CAF–immune cell interactions critically drive tumor immune evasion. Through TGF-β and IL-6 secretion, CAFs suppress CD8^+^ T-cell infiltration/function while recruiting regulatory T cells and myeloid-derived suppressor cells, establishing an immunosuppressive TME [58]. Furthermore, CAF heterogeneity confers context-dependent functions. Inflammatory CAFs predominantly secrete immunomodulatory factors, whereas myofibroblastic CAFs specialize in ECM remodeling[59]. In OSCC, ITGB2^+^CAFs activate the PI3K/AKT/mTOR axis via NADH oxidation during mitochondrial oxidative phosphorylation, accelerating tumor proliferation [60]. Additionally, PDPN^+^ CAFs transport the exosomal lncRNA FTX to tumor cells, activating the FTX–FEN1–ACSL4 axis to inhibit ferroptosis and potentiate invasiveness [61].

Multiple studies have established the pivotal role of the CCL7–CCR1 axis in facilitating metastasis across diverse cancers. Yang et al. demonstrated that CCL7 promotes osteoclast precursor migration via CCR1, the dominant factor in bone destruction during colorectal cancer metastasis [20]. In gastric cancer models, Chen et al. revealed that CCL7–CCR1 signaling activates the ERK/ELK1 pathway, drives SOX18 expression, and upregulates CCL7 to establish a positive feedback loop that enhances tumor invasiveness [18]. CCL7 is also significantly upregulated in rheumatoid arthritis, activating the JAK2–STAT1 pathway through CCR1 to form an autocrine feedback loop. This promotes M1 macrophage polarization while suppressing M2 polarization and exacerbates synovial inflammation [62]. Yu et al. discovered that CCR1 and its ligands (CCL7, MIP-1α, RANTES) enhance chemotactic recruitment of osteoclast precursors, potentiate RANKL-induced differentiation, and increase mature osteoclast mobility—all induced by inflammatory cytokines—highlighting CCR1’s critical role in inflammation-associated bone destruction [63]. Additionally, Jiao et al. reported that CAFs in hepatocellular carcinoma secrete CCL7, promoting tumor cell migration/invasion and activating TGF-β signaling to induce EMT, thereby driving distant metastasis [64]. The current study extends this paradigm by revealing that in OSCC, ZBP1 indirectly activates the CCR1 pathway in tumor cells by regulating CCL7 expression in CAFs, enhancing their proliferation, migration, and invasion. Moreover, contrasting CCL7’s predominant activation of TGF-β-induced EMT in hepatocellular carcinoma, CAF-derived CCL7 in OSCC primarily operated through CCR1 activation and downstream signaling. This establishes a tumor cell-sensing module for CAF-derived signals, ultimately driving pro-tumorigenic phenotypes.

This study has several limitations. The conclusions are predominantly derived from mouse models and murine MOC1 cell lines, rendering the applicability of these findings to human oral squamous cell carcinoma (OSCC) uncertain. In addition, although the research concentrated on the CCL7–CCR1 axis, it is plausible that ZBP1 may also modulate other chemokines, such as CXCLs, or influence other signaling pathways, including IL-1–related cascades. The comprehensive role of ZBP1 in tumor immunomodulation remains inadequately understood. Moreover, while BX471 was employed as a pharmacological inhibitor of CCR1 to elucidate the function of the CCL7–CCR1 axis, the possibility of off-target effects cannot be excluded. Future investigations utilizing CCR1 gene knockdown or more selective inhibitors are warranted to substantiate these findings [65]. Finally, the influence of ZBP1 on immune cells has not been extensively examined, underscoring the necessity for immune cell–specific conditional knockout models in subsequent research endeavors.

## Conclusion

This study elucidated the pro-tumorigenic role of ZBP1 in OSCC progression and delineated its core mechanism. ZBP1 orchestrates CAF-derived CCL7 secretion to activate the CCR1 receptor on tumor cells, enhancing their proliferative, migratory, and invasive capacities. By establishing a *Zbp1* knockout mouse model integrated with scRNA-seq and functional validation, ZBP1 was identified as a pivotal bridging molecule in CAF–tumor cell interactions, positioning it as a key regulator of the TME and a driver of OSCC development. In vitro and in vivo gain and loss-of-function experiments confirmed the functional dependence of the ZBP1–CCL7–CCR1 signaling axis, highlighting its potential as a therapeutic target.

## Supplementary information

Supplementary Table 1

Revised Supplemental Fig. 1

Revised Supplemental Fig. 2

Revised Supplemental Fig. 3

Revised Supplemental Fig. 4

Revised Supplemental Fig. 5

Revised Supplemental Fig. 6

Revised Supplemental Fig. 7

Revised Supplemental Fig. 8

supplement Materials-rss values.csv

original data

## Data Availability

All data, models, and code generated or used during the study appear in the submitted article.
