# Supplementary material for: Single-cell RNA sequencing identifies ZBP1-dependent mechanisms in OSCC progression
Source: Cell Death Dis. 2025 Dec 22;16(1):918. doi: 10.1038/s41419-025-08349-7 (PMC12749536; doi:10.1038/s41419-025-08349-7)
Supplement: Supplementary file 1 — Supplementary Table 1 [file 41419_2025_8349_MOESM1_ESM.docx]

**Table 1. Plasmid Construction and qRT-PCR Primer Sequences**

| Primer | Sequence (5’-3’) |
| --- | --- |
| sgZbp1-forward | CACCGTGAGCTATGACGGACAGACG |
| sgZbp1-reverse | AAACCGTCTGTCCGTCATAGCTCAC |
| sgNS-forward | CACCGTGCGAATACGCCCACGCGAT |
| sgNS-reverse | AAACATCGCGTGGGCGTATTCGCAC |
| Zbp1-forward | AAGAGTCCCCTGCGATTATTTG |
| Zbp1-reverse | TCTGGATGGCGTTTGAATTGG |
| α-SMA-forward | GTCCCAGACATCAGGGAGTAA |
| α-SMA-reverse | TCGGATACTTCAGCGTCAGGA |
| Fap-forward | TATCACCAGCGTAGCCTTTGA |
| Fap-reverse | CGATCAGGCAGACAACCATATAG |
| Col1a1-forward | ACGCCATCAAGGTCTACTGC |
| Col1a1-reverse | ACTCGAACGGGAATCCATCG |
| Ccl7-forward | GCTGCTTTCAGCATCCAAGTG |
| Ccl7-reverse | CCAGGGACACCGACTACTG |
| β-Actin-forward | GTGACGTTGACATCCGTAAAGA |
| β-Actin-reverse | GCCGGACTCATCGTACTCC |
