# Supplementary material for: Single-cell RNA sequencing identifies ZBP1-dependent mechanisms in OSCC progression
Source: Cell Death Dis. 2025 Dec 22;16(1):918. doi: 10.1038/s41419-025-08349-7 (PMC12749536; doi:10.1038/s41419-025-08349-7)
Supplement: Supplementary file 2 — Revised Supplemental Fig. 1 [file 41419_2025_8349_MOESM2_ESM.docx]

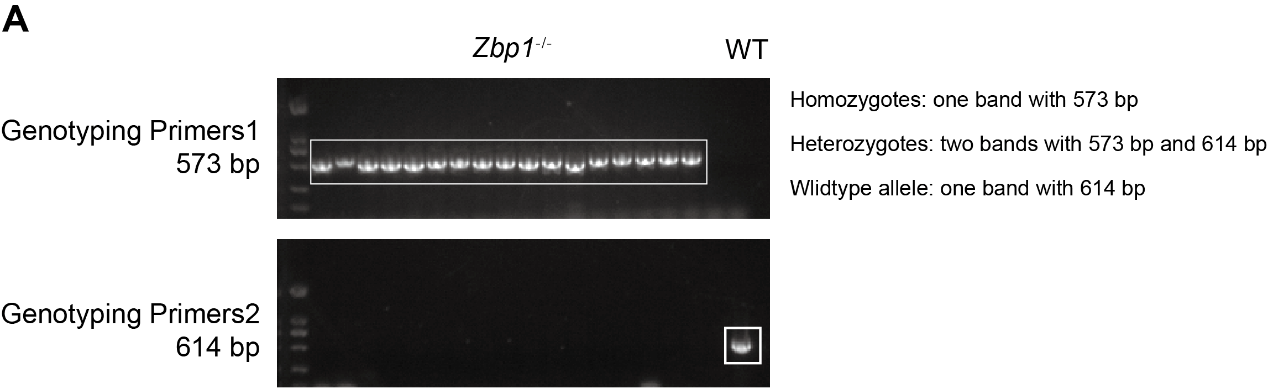


**Figure S1. Genotyping of mice.** (A) Genotypes of mice were identified by PCR of tail DNA followed by agarose gel electrophoresis.
