# Supplementary material for: Single-cell RNA sequencing identifies ZBP1-dependent mechanisms in OSCC progression
Source: Cell Death Dis. 2025 Dec 22;16(1):918. doi: 10.1038/s41419-025-08349-7 (PMC12749536; doi:10.1038/s41419-025-08349-7)
Supplement: Supplementary file 3 — Revised Supplemental Fig. 2 [file 41419_2025_8349_MOESM3_ESM.docx]

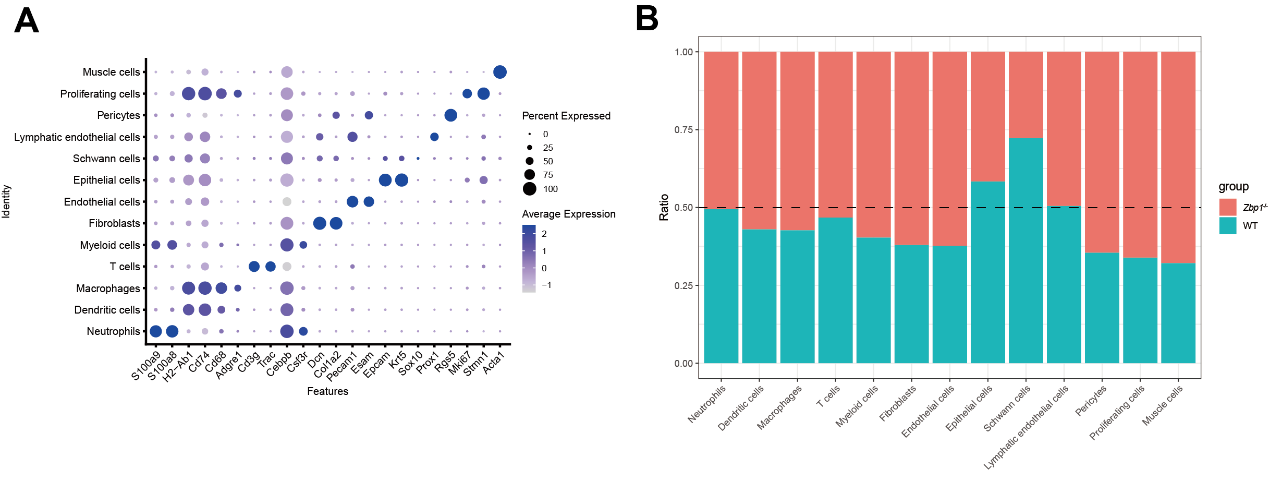


**Figure S2. Expression profiles and subset composition from single-cell RNA-seq.** (A) Expression patterns of selected biomarkers across different cell subsets. (B) Proportions of each identified cell subset.
