# Supplementary material for: Single-cell RNA sequencing identifies ZBP1-dependent mechanisms in OSCC progression
Source: Cell Death Dis. 2025 Dec 22;16(1):918. doi: 10.1038/s41419-025-08349-7 (PMC12749536; doi:10.1038/s41419-025-08349-7)
Supplement: Supplementary file 4 — Revised Supplemental Fig. 3 [file 41419_2025_8349_MOESM4_ESM.docx]

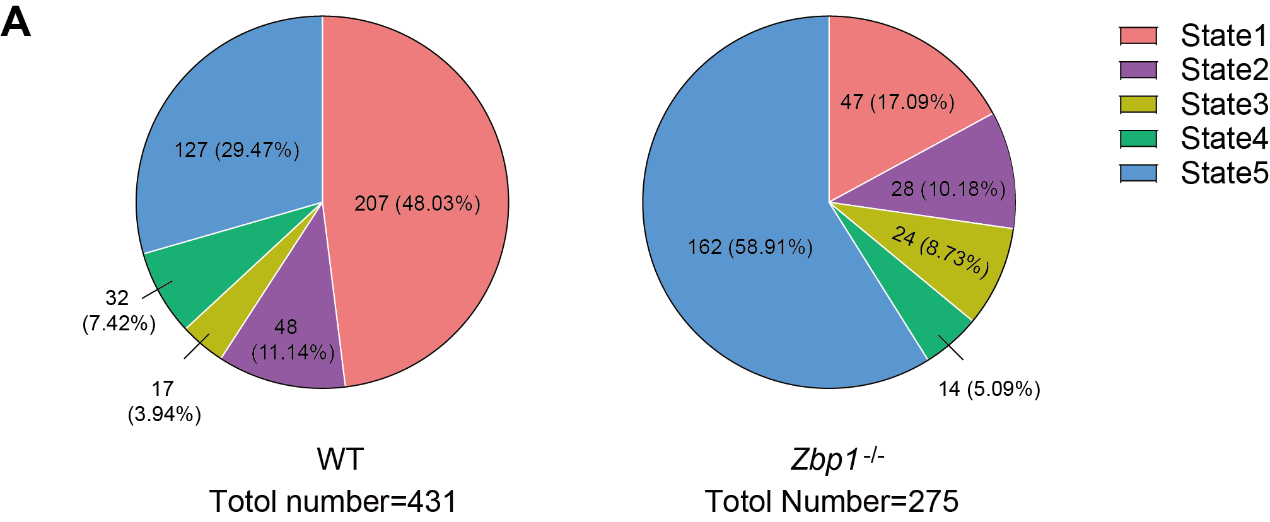


**Figure S3. Distribution of tumor cells in each state in WT and *Zbp1*^-/-^ groups.**

(A) Pie charts showing the distribution and proportion of tumor cells across five states (corresponding to those defined in Fig. 3B) in WT and *Zbp1*^-/-^ groups. Each slice represents the number and percentage of cells belonging to a specific state within the respective group. The total number of analyzed cells is indicated below each chart (WT: n = 431; *Zbp1*^-/-^: n = 275).
