# Supplementary material for: Single-cell RNA sequencing identifies ZBP1-dependent mechanisms in OSCC progression
Source: Cell Death Dis. 2025 Dec 22;16(1):918. doi: 10.1038/s41419-025-08349-7 (PMC12749536; doi:10.1038/s41419-025-08349-7)
Supplement: Supplementary file 5 — Revised Supplemental Fig. 4 [file 41419_2025_8349_MOESM5_ESM.docx]

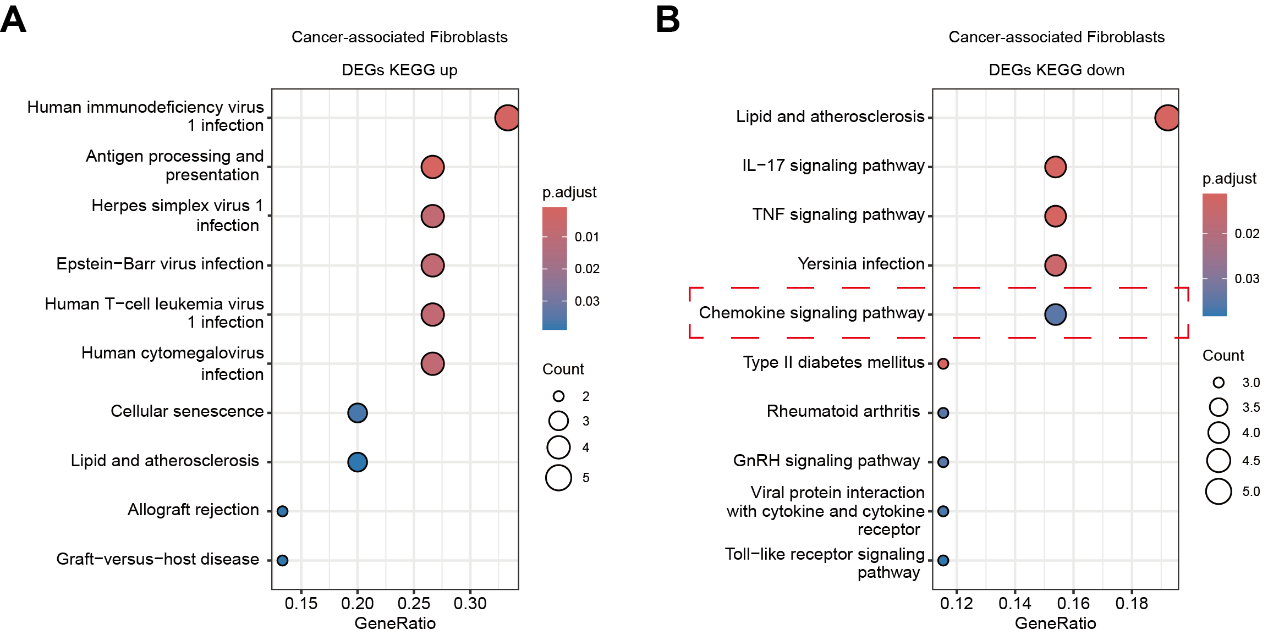


**Figure S4. KEGG enrichment analysis of differentially expressed genes in tumor-associated fibroblasts (CAFs).** (A) Pathways enriched by upregulated genes. (B) Pathways enriched by downregulated genes.
