# Supplementary material for: Single-cell RNA sequencing identifies ZBP1-dependent mechanisms in OSCC progression
Source: Cell Death Dis. 2025 Dec 22;16(1):918. doi: 10.1038/s41419-025-08349-7 (PMC12749536; doi:10.1038/s41419-025-08349-7)
Supplement: Supplementary file 7 — Revised Supplemental Fig. 6 [file 41419_2025_8349_MOESM7_ESM.docx]

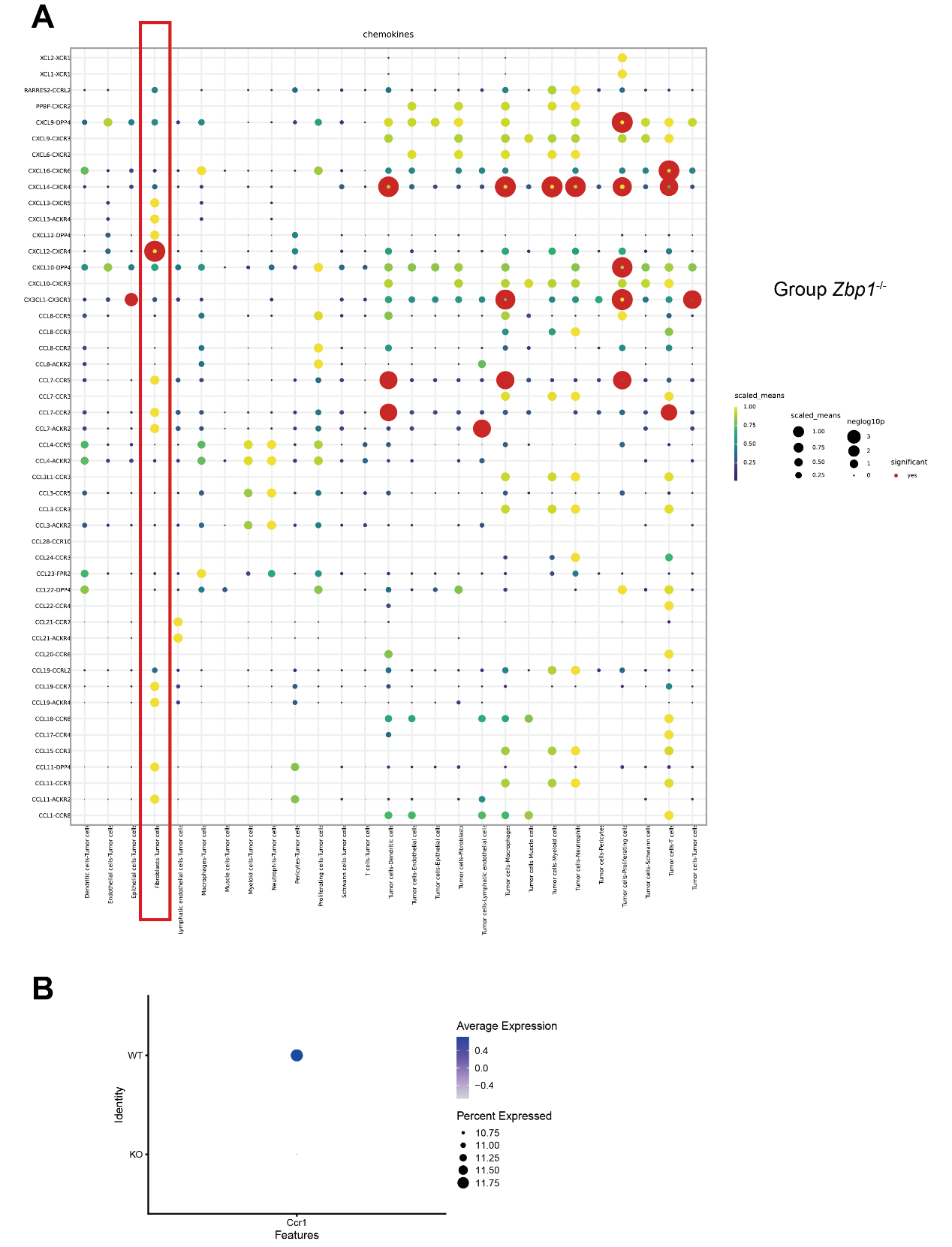


**Figure S6. Chemokine receptor–ligand interactions and Ccr1 expression in the *Zbp1*^-/-^ group.** (A) CellPhoneDB analysis of chemokine receptor–ligand interactions related to tumor cells in the *Zbp1*^-/-^ group. The plot displays all detected interactions under default thresholds, including both significant and non-significant pairs. Dot color represents scaled mean expression levels, while dot size reflects the statistical significance. Red circles highlight significant interactions. (B) Dot plot comparison of *Ccr1* expression between WT and *Zbp1*^-/-^ groups. Dot size indicates the percentage of cells expressing *Ccr1*, and color reflects the average expression level.
