# Supplementary material for: Single-cell RNA sequencing identifies ZBP1-dependent mechanisms in OSCC progression
Source: Cell Death Dis. 2025 Dec 22;16(1):918. doi: 10.1038/s41419-025-08349-7 (PMC12749536; doi:10.1038/s41419-025-08349-7)
Supplement: Supplementary file 8 — Revised Supplemental Fig. 7 [file 41419_2025_8349_MOESM8_ESM.docx]

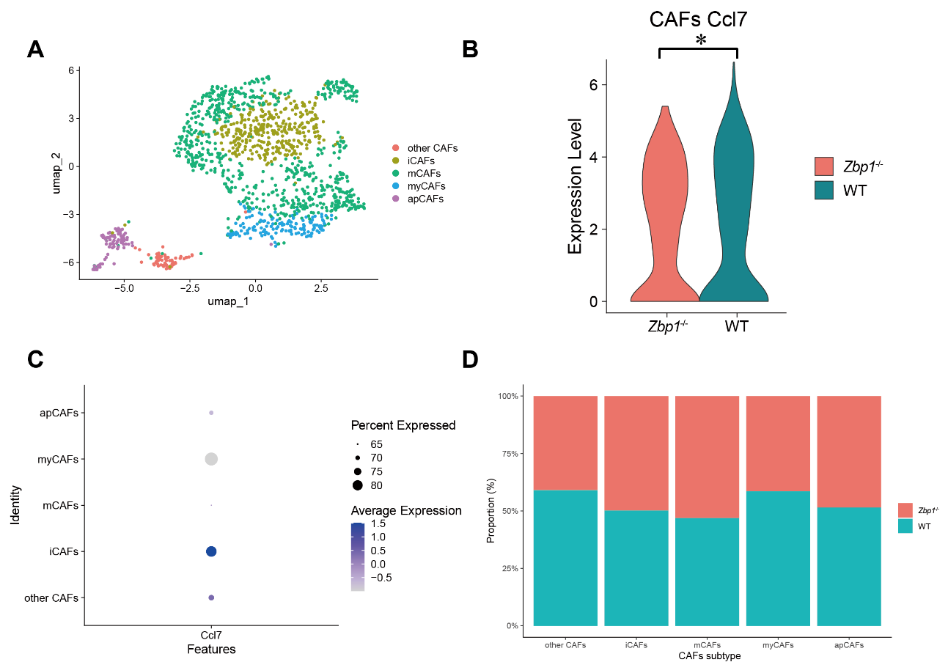


**Figure S7. Subcluster analysis of CAFs and Ccl7 expression.** (A) UMAP visualization showing further subclustering of CAFs into iCAFs, mCAFs, myCAFs, apCAFs, and other CAFs. (B) Violin plot showing the expression level of *Ccl7* in CAFs from WT and *Zbp1^-/-^* groups. (C) Dot plot analysis of *Ccl7* expression across CAF subclusters. Dot size represents the percentage of cells expressing *Ccl7*, and color indicates the average expression level. (D) Proportional distribution of CAF subclusters in WT and *Zbp1*^-/-^ groups.
