# Supplementary material for: Single-cell RNA sequencing identifies ZBP1-dependent mechanisms in OSCC progression
Source: Cell Death Dis. 2025 Dec 22;16(1):918. doi: 10.1038/s41419-025-08349-7 (PMC12749536; doi:10.1038/s41419-025-08349-7)
Supplement: Supplementary file 9 — Revised Supplemental Fig. 8 [file 41419_2025_8349_MOESM9_ESM.docx]

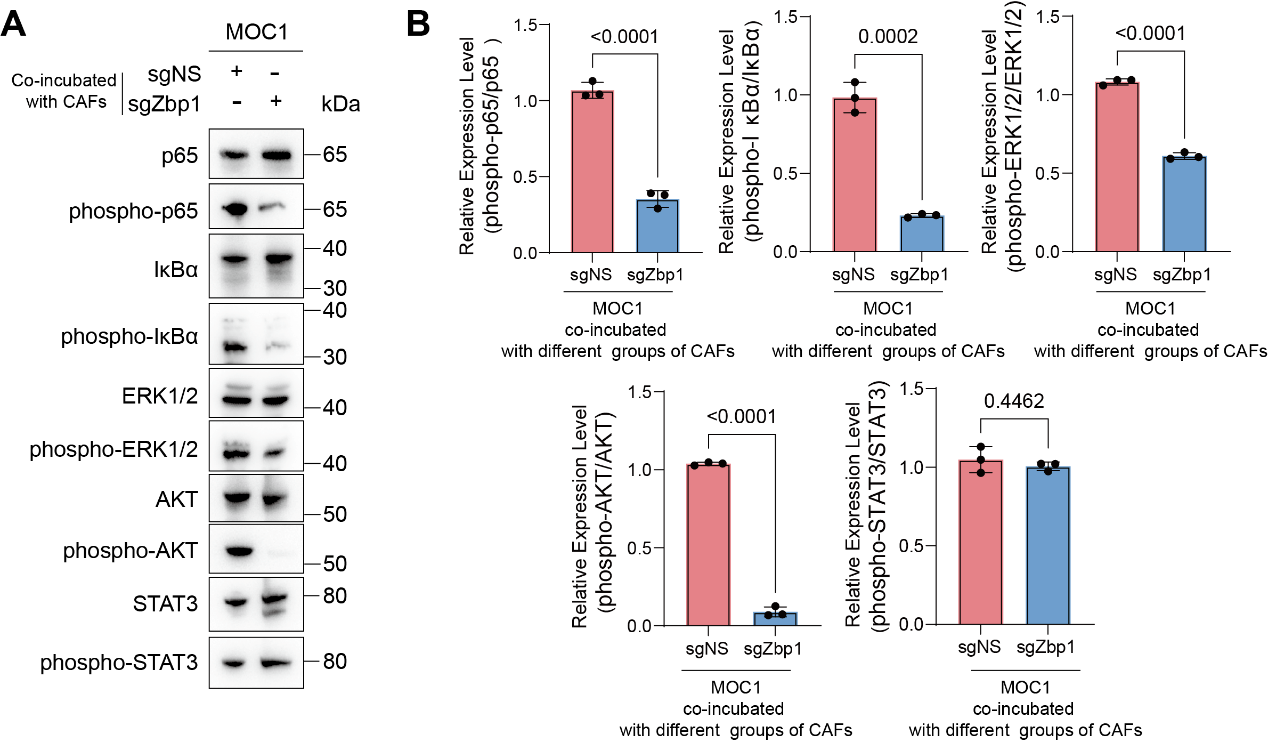


**Figure S8. Analysis of signaling pathways in MOC1 cells co-cultured with CAFs.** (A) Western blot analysis of NF-κB (p65, IκBα), ERK1/2, AKT, and STAT3 signaling pathways in MOC1 cells co-cultured with differently treated CAFs (sgNS or sgZbp1). (B) Quantification of Western blot results showing the relative phosphorylation levels of p65, IκBα, ERK1/2, AKT, and STAT3 in MOC1 cells co-cultured with differently treated CAFs (sgNS or sgZbp1). Data are presented as mean ± SEM, with p-values indicated.
