# Supplementary material for: Single-cell RNA sequencing identifies ZBP1-dependent mechanisms in OSCC progression
Source: Cell Death Dis. 2025 Dec 22;16(1):918. doi: 10.1038/s41419-025-08349-7 (PMC12749536; doi:10.1038/s41419-025-08349-7)
Supplement: Supplementary file 11 — original data [file 41419_2025_8349_MOESM11_ESM.docx]

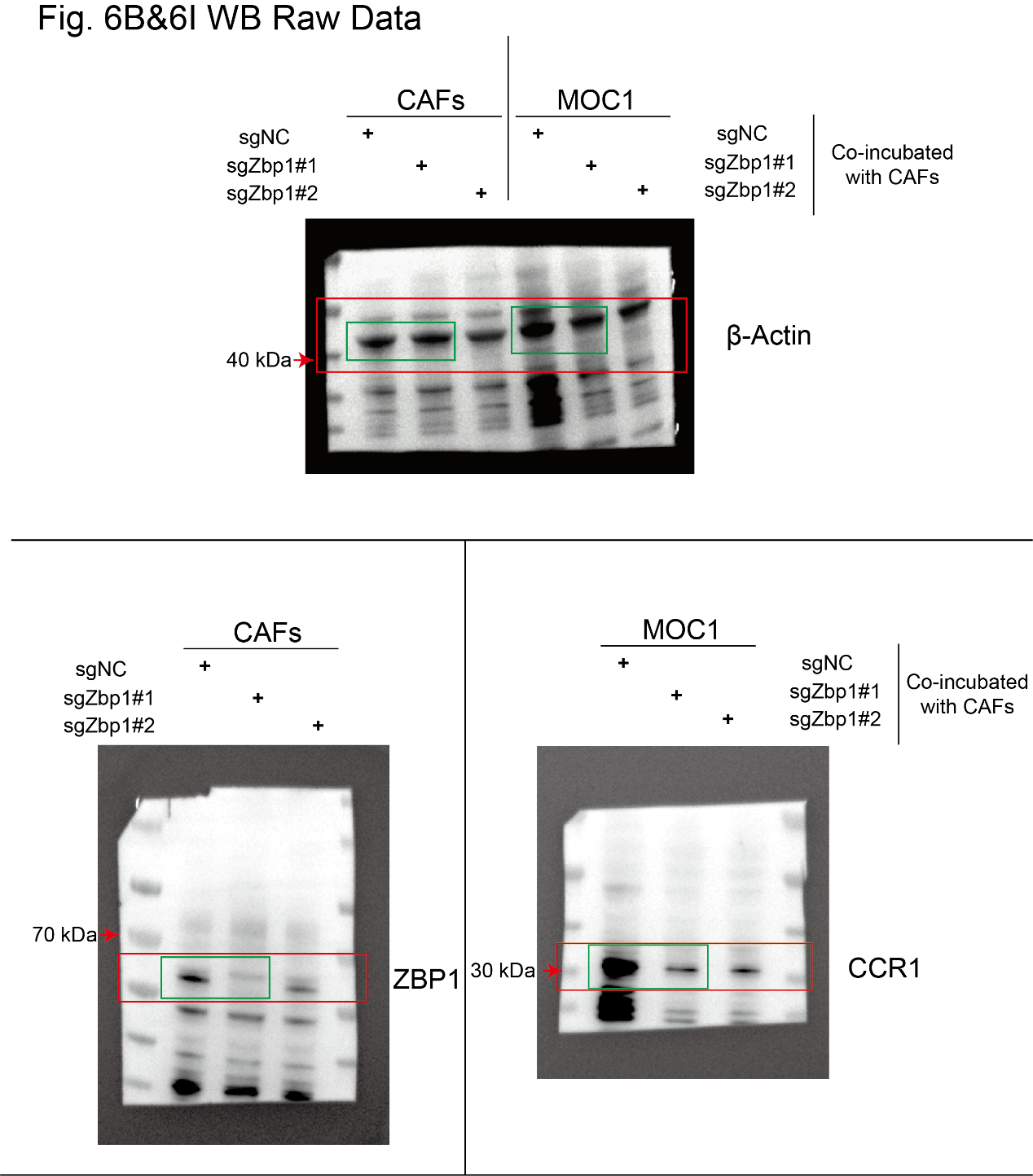


Fig. 6F p65 WB Raw Data


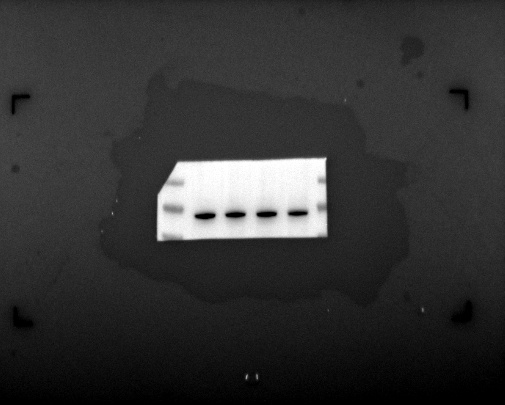


Fig. 6F p-p65 WB Raw Data


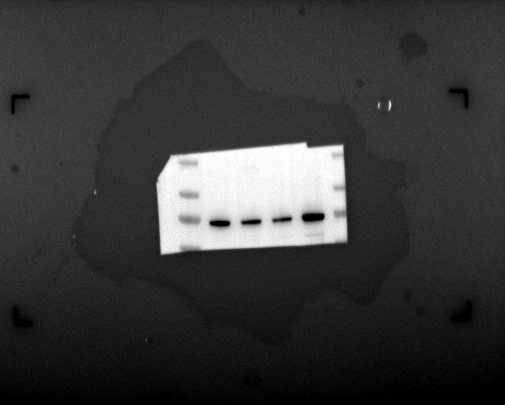


Fig. 6F IκBα WB Raw Data


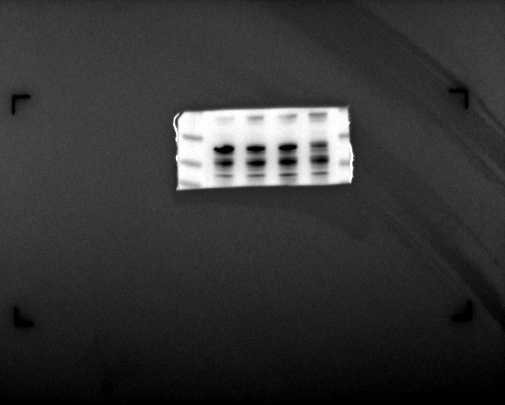


Fig. 6F p-IκBα WB Raw Data


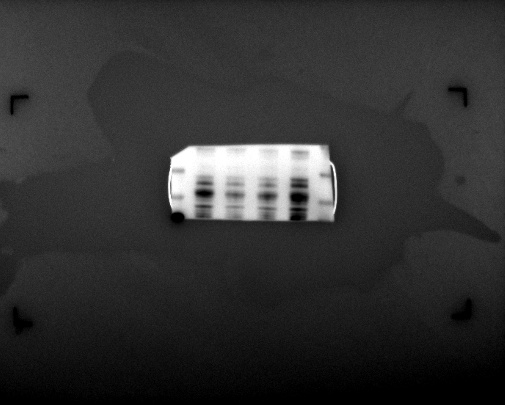


Fig. S3A p65 WB Raw Data


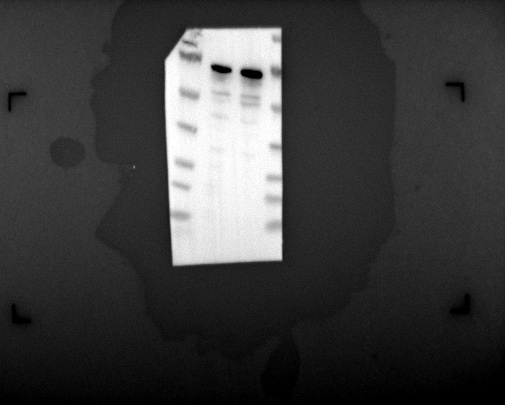


Fig. S3A p-p65 WB Raw Data


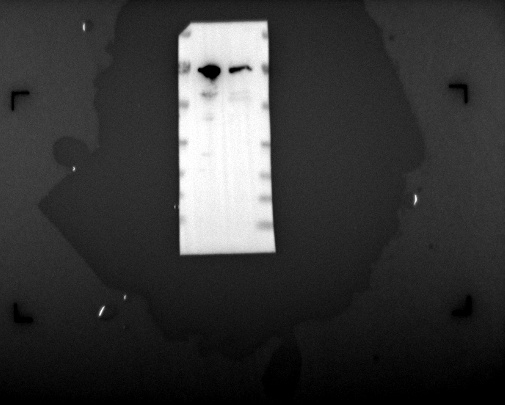


Fig. S3A IκBα WB Raw Data


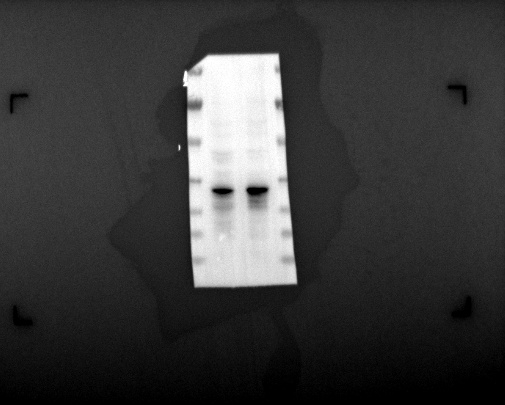


Fig. S3A p-IκBα WB Raw Data


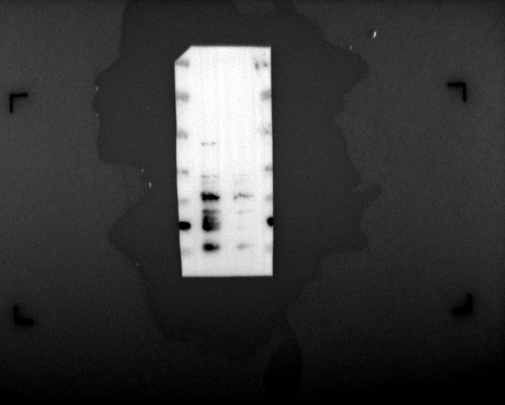


Fig. S3A ERK1/2 WB Raw Data


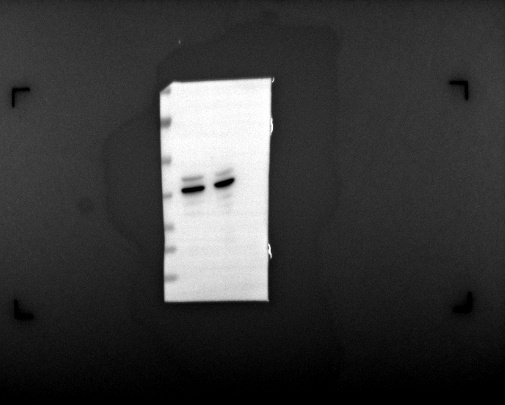


Fig. S3A p-ERK1/2 WB Raw Data


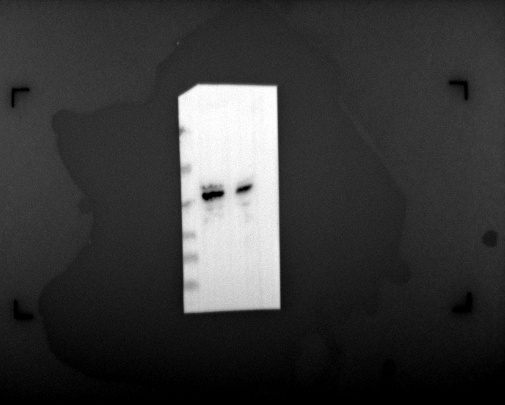


Fig. S3A AKT WB Raw Data


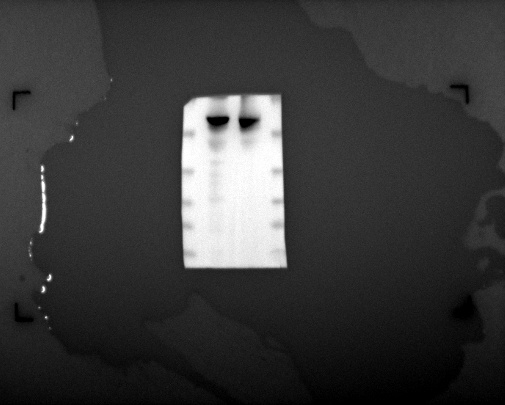


Fig. S3A p-AKT WB Raw Data


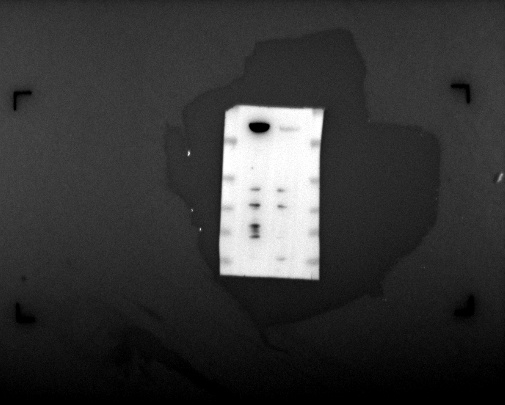


Fig. S3A STAT3 WB Raw Data


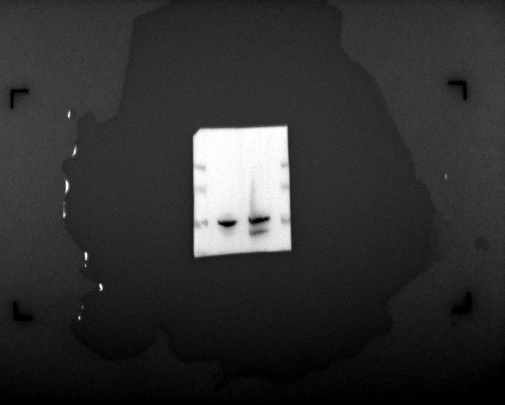


Fig. S3A p-STAT3 WB Raw Data


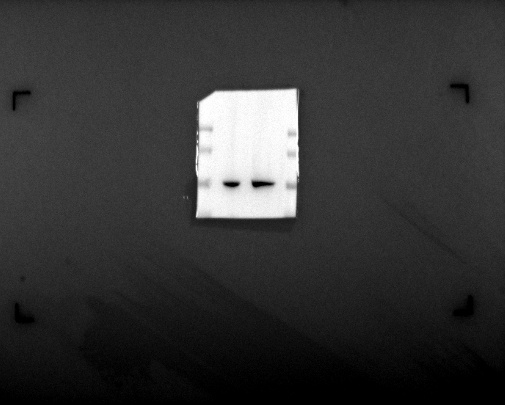


Fig. 5A qRT-PCR raw data

| Sample | Ct_1 | Ct_2 | Ct_3 |
| --- | --- | --- | --- |
| MEFs_β-Actin | 14.887 | 14.793 | 14.902 |
| MEFs_Acta2 | 18.496 | 18.582 | 18.551 |
| MEFs_Fap | 23.590 | 23.262 | 23.254 |
| MEFs_Col1a1 | 17.543 | 17.418 | 17.426 |
| CAFs_β-Actin | 16.996 | 17.074 | 17.012 |
| CAFs_Acta2 | 20.199 | 20.270 | 20.262 |
| CAFs_Fap | 25.160 | 24.879 | 25.191 |
| CAFs_Col1a1 | 19.270 | 19.160 | 19.043 |

Fig. 6A qRT-PCR raw data

| Sample | Ct_1 | Ct_2 | Ct_3 |
| --- | --- | --- | --- |
| CAFs sgNS_β-Actin | 14.730 | 14.645 | 14.777 |
| CAFs sgNS_Zbp1 | 22.191 | 21.965 | 22.129 |
| CAFs sgZbp1_β-Actin | 14.707 | 14.770 | 14.777 |
| CAFs sgZbp1_Zbp1 | 24.324 | 24.277 | 24.629 |

Fig. 6C qRT-PCR raw data

| Sample | Ct_1 | Ct_2 | Ct_3 |
| --- | --- | --- | --- |
| CAFs sgNS_β-Actin | 14.395 | 14.355 | 14.363 |
| CAFs sgNS_Ccl7 | 21.660 | 21.676 | 21.613 |
| CAFs sgzbp1_β-Actin | 14.637 | 14.824 | 14.480 |
| CAFs sgzbp1_Ccl7 | 23.293 | 23.074 | 23.012 |
